# FUNDC1 Attenuates UVA-Induced Skin Photoaging by Regulating Mitophagy and P53 Stability

**DOI:** 10.3390/antiox15091063

**Published:** 2026-08-25

**Authors:** Chang Zhang, Menghui Hou, Nan Wang, Yiqiong Liang, Qianhui Ma, Minghe Li, Yixiao Zhang, Haiying Zhang, Yingai Shi, Huimei Yu, Xu He

**Affiliations:** Department of Pathololgy, College of Basic Medical Sciences, The Medical Basic Research Innovation Center of Airway Disease in North China, Key Laboratory of Pathobiology, Ministry of Education, Jilin University, Changchun 130021, China; zhangchang24@mails.jlu.edu.cn (C.Z.); houmh24@mails.jlu.edu.cn (M.H.); nwang24@mails.jlu.edu.cn (N.W.); yiqiong25@mails.jlu.edu.cn (Y.L.); qhma23@mails.jlu.edu.cn (Q.M.); mhli22@mails.jlu.edn.cn (M.L.); yxzhang7017@163.com (Y.Z.); shiya@jlu.edu.cn (Y.S.);

**Keywords:** skin photoaging, FUNDC1, miR-137-3p, BAZ1B, P53 ubiquitination

## Abstract

Skin photoaging resulting from chronic ultraviolet A (UVA) exposure is closely associated with mitochondrial dysfunction and impaired cellular homeostasis. Mitophagy is an important mitochondrial quality control process, but the role of FUNDC1-associated mitophagy-related activity in skin photoaging remains incompletely understood. Here, we investigated the function and regulatory mechanisms of FUNDC1 in UVA-induced photoaging models. FUNDC1 expression was reduced in UVA-exposed human dermal fibroblasts (HDFs) and in photoaged mouse skin. FUNDC1 knockdown aggravated photoaging-associated phenotypes, mitochondrial dysfunction, and altered autophagy/mitophagy-related activity, whereas FUNDC1 overexpression attenuated these changes in vitro and in vivo. Pharmacological modulation further showed that Rapa partially counteracted FUNDC1 knockdown-associated effects, while Mdivi-1 weakened the protective effects associated with FUNDC1 overexpression, supporting the involvement of mitophagy-related mitochondrial quality control. Mechanistically, miR-137-3p was upregulated during UVA-induced photoaging and negatively regulated FUNDC1 expression through the predicted FUNDC1 3′UTR binding site. In addition, FUNDC1 was concerned with proteasome-dependent regulation of P53 protein stability. BAZ1B was identified as a candidate P53-associated ubiquitination regulator that participated in FUNDC1-associated regulation of P53 ubiquitination and stability. LC-MS/MS analysis combined with site-directed mutagenesis further manifested that P53 K292 was a major ubiquitination site involved in BAZ1B-associated regulation of P53 stability. In vivo, BAZ1B knockdown attenuated FUNDC1-associated protection against UVA-induced skin photoaging and reduced P53 ubiquitination. Collectively, these findings indicate that FUNDC1 can attenuate UVA-induced skin photoaging by preserving mitophagy-related mitochondrial homeostasis and modulating BAZ1B-associated P53 stability, with miR-137-3p acting as an upstream negative regulator of FUNDC1.

## 1. Introduction

Skin photoaging is primarily caused by chronic ultraviolet (UV) exposure and is characterized by epidermal thickening, dermal collagen degradation, wrinkle formation, pigmentation, and impaired skin structure. Among the UV spectrum, ultraviolet A (UVA) accounts for the majority of solar UV radiation reaching the earth’s surface and can penetrate into the dermis, where it directly affects dermal fibroblasts and extracellular matrix homeostasis [1,2,3]. Human dermal fibroblasts (HDFs) are major cellular components of the dermis and play essential roles in collagen production, matrix remodeling, and skin structural maintenance. Therefore, elucidating the molecular mechanisms underlying UVA-induced HDF dysfunction is crucial for understanding and preventing skin photoaging.

Mitochondrial dysfunction has emerged as a key contributor to UV-induced cellular senescence and dermal degeneration [4,5]. Because mitochondria are highly susceptible to oxidative stress, efficient mitochondrial quality control is required to maintain cellular homeostasis under stress conditions. Mitophagy, a selective form of autophagy that removes damaged mitochondria, is an important component of mitochondrial quality surveillance [6,7]. FUN14 domain-containing 1 (FUNDC1) is a mitochondrial outer membrane receptor involved in mitophagy regulation and has been implicated in several pathological contexts associated with mitochondrial dysfunction [8]. However, whether FUNDC1 participates in UVA-induced skin photoaging and how altered FUNDC1 expression influences mitochondrial homeostasis in HDFs remains unclear.

MicroRNAs (miRNAs) are small non-coding RNAs that regulate gene expression post-transcriptionally and participate in diverse biological processes, including cellular senescence, metabolism and stress responses [9]. Increasing evidence suggests that miRNAs are involved in skin development, homeostasis, wound repair, and photoaging [10,11,12]. Previous studies have reported altered miRNA expression profiles in UVA-induced photoaged HDFs, suggesting that miRNA-mediated gene regulation may contribute to fibroblast dysfunction during photoaging. Notably, miR-137 has been reported to regulate mitophagy under hypoxic conditions by targeting the mitophagy receptors FUNDC1 and NIX [13]. However, whether miR-137-3p is altered during UVA-induced skin photoaging and whether miR-137-3p exerts an impact on FUNDC1 expression in this context remains to be determined.

P53 signaling is a central regulator of cellular senescence, stress responses, and cell fate decisions as well [14,15,16]. Autophagy and mitochondrial homeostasis are closely linked to P53 signaling, and dysregulated P53 activity can contribute to senescence-associated phenotypes [17,18]. P53 protein abundance is tightly controlled by post-translational mechanisms, particularly ubiquitin-proteasome-mediated degradation. E3 ubiquitin ligases are critical determinants of substrate specificity in the ubiquitination cascade [19]. In this study, pathway enrichment analysis highlighted P53 signaling as a candidate pathway associated with autophagy-related gene alterations in UVA-induced photoaged HDFs. We therefore explored whether FUNDC1 is associated with post-transcriptional regulation of P53 protein stability.

Zinc finger domain 1B (BAZ1B), also known as WSTF, is a chromatin-related protein involved in transcriptional regulation, DNA damage responses, and chromatin remodeling. Bioinformatic prediction suggested that BAZ1B may be a candidate P53-associated ubiquitination regulator. Nevertheless, the potential relationship among FUNDC1, BAZ1B and P53 stability in skin photoaging has not been established. Given the distinct subcellular localization and functions of FUNDC1 and BAZ1B, whether and how FUNDC1-associated mitochondrial quality control is linked to BAZ1B/P53 signaling requires further investigation.

In the present study, UVA-induced HDF and mouse skin photoaging models were first established to investigate the role of FUNDC1 in photoaging and related mitochondrial dysfunction. Furthermore, the regulatory effect of miR-137-3p on FUNDC1 expression and function was explored. We also investigated the involvement of BAZ1B in FUNDC1-associated regulation of P53 ubiquitination and stability. The novelty of the present study lies in extending the role of FUNDC1 beyond mitophagy-related mitochondrial quality control by linking FUNDC1-associated changes to the regulation of P53 protein stability in UVA-induced photoaging. In addition, our study identifies BAZ1B as a candidate regulator involved in this process and further implicates P53 K292 as a major ubiquitination site associated with BAZ1B-related regulation of P53 stability. Our findings support a regulatory model involving miR-137-3p, FUNDC1, mitophagy-related mitochondrial quality control, and BAZ1B/P53 signaling in UVA-induced skin photoaging.

## 2. Materials and Methods

### 2.1. Reagents and Materials

Rapamycin (HY-10219), MG-132 (HY-13259), Baf-A1 (HY-100558), Mdivi-1 (HY-15886), Tween 80 (HY-Y1891) and PEG300 (HY-Y0873) were obtained from MedChemExpress (MCE, Monmouth Junction, NJ, USA). The lentivirus overexpressing FUNDC1 in cells and the adenovirus overexpressing FUNDC1 and knocking down BAZ1B in animals were derived from Gikai Gene (Shanghai, China). Cell culture dishes and plates were provided by NEST Biotechnology (Wuxi, China).

### 2.2. Isolation, Culture and Characterization of HDFs

Primary HDFs were isolated from circumcised foreskins of healthy human donors aged 5 to 20 years. The isolated cells were digested with neutral protease and type I collagenase (Yeasen, Shanghai, China) (Supplementary Figure S6A) and then cultured in high-glucose DMEM medium (Sevenbio, Beijing, China) supplemented with 10% fetal bovine serum (BaiDi Biotechnology Co., Ltd. (BDBIO), Shenzhen, China) and 1% penicillin–streptomycin (A200-100, BDBIO, Hangzhou, China) at 37 °C in a 5% CO_2_ incubator. All primary HDFs were obtained following written informed consent from donors, in accordance with a protocol approved by the Institutional Review Board of China-Japan Union Hospital of Jilin University. Microscopic observations of passage 1 (P1) and passage 4 (P4) cells exhibited a spindle-shaped morphology with clear boundaries. When cells reached 90% confluence, they displayed a swirling pattern (Supplementary Figure S6B). Immunofluorescence staining of P4 cells revealed high expression of the mesenchymal marker Vimentin with a positivity rate of over 97%, and no expression of the epithelial marker E-cadherin (Supplementary Figure S6C), confirming that the isolated cells were dermal fibroblasts and suitable for further experiments.

### 2.3. Immunofluorescence Staining

Cells were cultured on glass coverslips within 24-well culture plates. After washing with PBS, cells were fixed with 4% paraformaldehyde for 10 min at room temperature (RT), permeabilized with 0.1% Triton X-100 for 15 min, and blocked with 5% BSA for 30 min. Then cells were incubated overnight at 4 °C with primary antibodies against Vimentin (5741, CST, Danvers, MA, USA) and E-cadherin (3195, CST, Danvers, MA, USA), followed by 1 h incubation at RT with DyLight 488-conjugated (AS037, Abclonal, Wuhan, China) and DyLight 594-conjugated (AS039, Abclonal, Wuhan, China) secondary antibodies. Nuclei were counterstained with DAPI (G1407-25ML, Servicebio, Wuhan, China) for 5 min at RT.

### 2.4. Preparation of HDF Photoaging Models

Photoaging in human dermal fibroblasts (HDFs) was induced through UVA irradiation. HDFs were cultured until 50% confluence was reached, followed by daily irradiation with UVA (5 J/cm^2^) for three consecutive days in PBS. After irradiation, cells were maintained in complete medium for an additional 24 h prior to analysis.

### 2.5. 5-Ethynyl-2′-deoxyuridine (EDU) Incorporation Assay

The EDU incorporation assay was performed using the Cell-Light EDU Apollo 488 In Vitro Imaging Kit (C10310, Beyotime Biotechnology, Shanghai, China) in strict accordance with the manufacturer’s protocol. Fluorescent images were acquired using an Olympus DP70 microscope (Olympus Corporation, Tokyo, Japan) equipped with a 20× objective lens. EDU-positive cells were quantified in five randomly selected fields per sample using ImageJ 1.54f for subsequent statistical analysis.

### 2.6. Senescence-Associated β-Galactosidase (SA-β-gal) Staining

HDFs were seeded in 6-well plates at a density of 2 × 10^5^ cells per well. Upon reaching 70% confluence, cells were washed three times with PBS. SA-β-gal activity was assessed using a senescence-associated β-galactosidase staining kit (C0602, Beyotime Biotechnology, Shanghai, China). Cells were firstly fixed with 500 μL of 4% paraformaldehyde per well for 15 min at RT, followed by three PBS washes. The staining solution was prepared by mixing 10 μL of solution A, 10 μL of solution B, 930 μL of solution C, and 50 μL of X-Gal (20 mg/mL in DMSO), with 1 mL of the working solution added to each well. Plates were light-protected with aluminum foil and incubated in a humidified chamber at 37 °C overnight (16–18 h) without CO_2_. SA-β-gal-positive cells (exhibiting blue cytoplasmic staining) were quantified under phase-contrast microscopy (Olympus IX73, Tokyo, Japan) by counting at least 500 cells per sample across five random fields, with positive rates calculated using ImageJ software.

### 2.7. Gene Expression Analysis

Total RNA was extracted from HDFs using Trizol reagent (Takara, Beijing, China) and reverse-transcribed into cDNA. RT-qPCR was performed using an ABI 7300 Real-Time PCR System with SYBR Green. The specific primer sequences are listed in Table 1. The thermal cycling protocol included pre-incubation at 95 °C for 2 min, followed by 40 cycles at 95 °C for 15 s, annealing at 60 °C for 30 s, and a final extension at 72 °C for 5 min. β-actin was used as the reference gene for normalizing mRNA expression via the 2^−ΔΔCt^ method.

### 2.8. Western Blot Analysis

Total protein was extracted using RIPA lysis buffer (P0013B, Beyotime, Shanghai, China) with protease inhibitor. Protein concentration was measured using a BCA Protein Assay Kit (KTD3001, Abbkine, Wuhan, China). Protein samples (50 μg) were then separated by SDS-PAGE, transferred to PVDF membranes (Burlington, MA, USA) and blocked with 5% non-fat milk. Following blocking and prior to primary antibody incubation, the PVDF membranes were physically sectioned horizontally into strips according to the expected molecular weights of the target proteins, with reference to the molecular weight markers. The individual membrane strips were subsequently incubated with the corresponding primary antibodies. Membranes were further incubated overnight at 4 °C with the following primary antibodies: MMP1 (1:1000, ZENBIO, Chengdu, China), Collagen-I (1:1000, ZENBIO, Chengdu, China), FUNDC1 (1:1000, ABclonal, Wuhan, China), LC3 (1:1000, ZENBIO, Chengdu, China), P62 (1:1000, ZENBIO, Chengdu, China), P53 (1:1000, ZENBIO, Chengdu, China), and BAZ1B (1:1500, P12428, ProMab Biotechnologies Inc., Richmond, CA, USA). After incubation with anti-rabbit IgG secondary antibodies, blots were visualized using an ECL detection system (Tanon, Shanghai, China) and quantified using ImageJ software.

### 2.9. Mitophagy Specificity Assay

Mitophagy was assessed using the Mtphagy Dye (MD01, Dojindo Laboratories, Kumamoto, Japan) following the manufacturer’s protocol with minor modifications. Cells were loaded with 100 nM Mtphagy Dye working solution for 30 min at 37 °C, washed twice with PBS, and then counterstained with 50 nM Lyso Dye for 15 min. Fluorescent images were obtained using a Zeiss LSM 880 confocal microscope (Oberkochen, Germany) with a 63× oil immersion objective.

### 2.10. Measurement of ROS Levels

Intracellular ROS levels were detected by incubating 5 × 10^3^ cells/well in a 24-well plate with 5 μM dihydroethidium (DHE, S0063, Beyotime Biotechnology, Shanghai, China) in serum-free medium for 30 min at 37 °C in the dark. After PBS washing, DHE fluorescence was immediately measured using a microscope and quantified using ImageJ software.

### 2.11. Determination of ATP Content

Cellular ATP levels were quantified using the ATP Assay Kit (S0026, Beyotime Biotechnology) according to the manufacturer’s instructions, with luminescence measured within 10 min of reagent addition. Chemiluminescence was recorded using a GloMax Navigator luminometer (Promega, Madison, WI, USA) with 1 s integration time, and ATP concentrations were determined against a freshly prepared ATP standard curve (0.1–10 μM).

### 2.12. FUNDC1 Knockdown or Overexpression in HDFs

For FUNDC1 knockdown, HDFs at 60–70% confluence were transfected with 50 nM FUNDC1-targeting siRNA using the RiboFECT^TM^ CP Transfection Kit (C10511, Beyotime, Shanghai, China) at a 1:3 (*w*/*v*) siRNA: reagent ratio, based on the manufacturer’s protocol. The si-FUNDC1 sequence was designed to target the coding sequence (CDS) of FUNDC1 mRNA, which is distinct from the miR-137-3p binding site located within the FUNDC1 3′UTR. For FUNDC1 overexpression, HDFs were infected with lentiviral particles (1 × 10^8^ TU/mL) carrying the pLVX-FUNDC1 or control vector (Gikai Gene, Shanghai, China) at a multiplicity of infection (MOI) of 20 in the presence of 5 μg/mL HitransG A/P transduction enhancer (LV063, Gikai Gene, Shanghai, China). Cells were maintained in complete medium for 72 h post-infection before analysis.

### 2.13. Dual-Luciferase Reporter Assay

A luciferase reporter assay was carried out using the psiCheck2 vector containing the full-length FUNDC1 3′ UTR. Luciferase activity was normalized to Renilla luciferase, and enzymatic activity was measured using a dual-luciferase reporter assay system.

### 2.14. Transfection with miR-137-3p Mimic and Inhibitor

HDFs at 70% confluence were transfected with 50 nM miR-137-3p mimic, inhibitor, or respective negative controls (RiboBio, Guangzhou, China) using the RiboFECT^TM^ CP Transfection Kit (C10511-05, RiboBio, Guangzhou, China), with transfection efficiency monitored by Cy3-labeled oligos. Total RNA was isolated 48 h post-transfection using Trizol reagent (15596026, Thermo Fisher, Waltham, MA, USA) and reverse-transcribed into cDNA for subsequent qPCR analysis of target genes.

### 2.15. Co-Immunoprecipitation (Co-IP)

Cells were lysed in ice-cold Co-IP buffer supplemented with 1× protease inhibitor cocktail (HY-K0010, MCE) and 1× phosphatase inhibitor cocktail (HY-K0022, MCE, Shanghai, China). After centrifugation at 12,000× *g* for 10 min at 4 °C, cleared lysates were incubated overnight at 4 °C with Protein A/G Magnetic Beads (L-1004, Biolinkedin, Shanghai, China) pre-conjugated with 2 μg of target-specific antibodies. Beads were washed five times with IP wash buffer, with 5 min rotations between washes. Immunocomplexes were eluted by boiling in 1 × SDS loading buffer containing 5% β-mercaptoethanol at 95 °C for 10 min.

### 2.16. P53 Ubiquitination Assay

Cells were lysed in immunoprecipitation buffer supplemented with protease inhibitors and N-ethylmaleimide. Equal amounts of cell lysates were incubated with an anti-P53 antibody overnight at 4 °C, followed by incubation with protein A/G agarose beads. After extensive washing, the immunoprecipitated proteins were eluted in SDS loading buffer and subjected to Western blot. Ubiquitinated P53 was detected using an anti-ubiquitin antibody, and immunoprecipitated P53 was examined using an anti-P53 antibody.

### 2.17. Cycloheximide Chase Assay

Cells were treated with cycloheximide (CHX, 10 μg/mL) to inhibit de novo protein synthesis. Cells were collected at the indicated time points and lysed for Western blot analysis. Target protein levels were normalized to the corresponding loading control and further normalized to the level at 0 h to evaluate protein degradation.

### 2.18. LC-MS for Ubiquitination Detection

HEK293T cells were transfected with the FLAG-P53 plasmid for 24 h and then exposed to MG132 for 24 h to inhibit proteasomal degradation. Cell lysates were treated with 1% sodium dodecyl sulfate (SDS) before immunoprecipitation using an antibody targeting the FLAG epitope tag. Then SDS-PAGE was performed, followed by Coomassie blue staining and the excision of bands for detection. Mass spectrometry (LTQ Orbitrap Elite, Thermo Fisher Scientific) was used to identify the ubiquitination of the P53 protein.

### 2.19. Establishment of Mouse Models of Skin Photoaging and Treatment

Female Kunming mice were irradiated with UVA at 20 J/cm^2^, 5 times weekly for 6 consecutive weeks. Following one week of acclimation under specific pathogen-free conditions, mice received intradermal injections of adeno-associated virus (AAV9, 1 × 10^12^ vg/mL in PBS) at five sites on the dorsal skin. Mdivi-1 treatment commenced 72 h post-initial irradiation, administered by intraperitoneal injection (20 mg/kg in 5% DMSO/30% PEG300/saline) five times weekly for six weeks, with vehicle controls receiving solvent alone.

### 2.20. Hematoxylin–Eosin (HE) Staining

Paraffin-embedded tissue sections were deparaffinized in fresh xylene twice for 30 min each, followed by dehydration in absolute ethanol twice for 5 min each. After hydration through graded ethanol to distilled water, sections were rinsed in running tap water and stained with hematoxylin for 10 min to visualize cell nuclei, then washed twice in tap water. Sections were differentiated in 1% hydrochloric acid ethanol for 10 s, rinsed twice in tap water, blued in weak ammonia water for 1 min, and finally rinsed again in tap water. Following dehydration through graded ethanol, sections were cleared in xylene and mounted with neutral balsam. Images were captured using a digital image acquisition system, with five random fields selected from each section.

### 2.21. Masson’s Trichrome Staining

Paraffin sections were deparaffinized and rehydrated through a standard series of xylene and ethanol. Sections were stained with hematoxylin for 10 min and washed in running tap water. Differentiation was performed briefly with 1% hydrochloric acid in ethanol, followed by thorough washing in running tap water for bluing. Subsequently, sections were immersed in Ponceau S-acid fuchsin solution for 10 min and rinsed in 0.2% acetic acid. The sections were then treated with 1% phosphomolybdic acid solution for 2 min and rinsed again in 0.2% acetic acid. This was followed by staining with 2% aniline blue solution for 5 min and a final rinse in 0.2% acetic acid. After dehydration through a graded ethanol series and clearing in xylene, the sections were mounted with neutral balsam for microscopic observation.

### 2.22. Immunohistochemistry (IHC)

For IHC detection of MMP-1, Collagen-I, FUNDC1, BAZ1B and P53 expression, deparaffinization and hydration were performed as above, followed by antigen retrieval in citrate buffer using pressure heating. Sections were rinsed three times in PBS (3 min each) and incubated with endogenous peroxidase blocking reagent at RT for 10 min, then rinsed again three times in PBS. Non-specific binding was blocked by incubation with blocking solution for 10 min at RT. Primary antibodies (diluted 1: 500) were applied, and sections were incubated overnight at 4 °C. The next day, sections were rinsed in PBS (three times, 3 min each) and incubated with biotinylated goat anti-mouse/rabbit IgG secondary antibody for 10 min at RT, followed by PBS washes. Staining was developed with freshly prepared diaminobenzidine (DAB) solution under microscopic monitoring to control reaction time. After rinsing in tap water, nuclei were counterstained with hematoxylin for 1 min and blued in running tap water. Finally, sections were dehydrated, cleared, and mounted with neutral balsam. Images were acquired for subsequent quantitative analysis.

### 2.23. Statistical Analysis

All statistical analyses were performed using GraphPad Prism 8.0 (GraphPad Software, San Diego, CA, USA). Data are presented as the mean ± standard deviation (SD). Comparisons between two independent groups were performed using unpaired two-tailed Student’s *t*-tests. Comparisons among multiple groups were performed using one-way or two-way analysis of variance (ANOVA), followed by Tukey’s multiple-comparisons test. The specific statistical test used for each experiment is indicated in the corresponding figure legend. A *p* value < 0.05 was considered statistically significant.

## 3. Results

### 3.1. UVA-Induced Photoaging Is Accompanied by Fundc1 Downregulation and Impaired Mitochondrial Homeostasis

**Figure 1:**
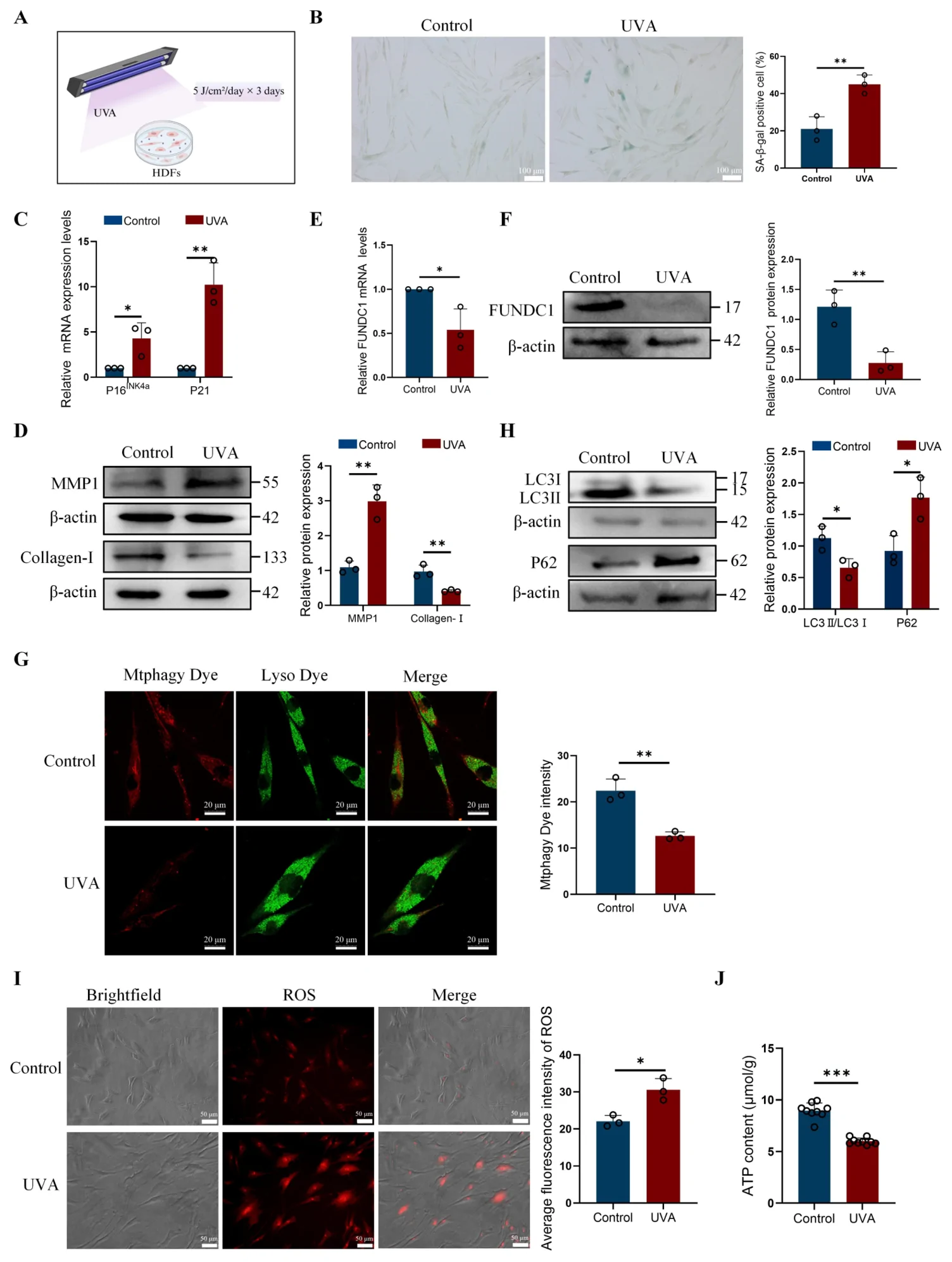
FUNDC1 is downregulated in UVA-induced photoaged HDFs and is associated with impaired mitochondrial homeostasis. (**A**) Schematic illustration of the UVA-induced HDF photoaging model. (**B**) Representative SA-β-gal staining images and quantification of SA-β-gal-positive cells in control and UVA-exposed HDFs. (**C**) RT-qPCR analysis of the senescence-associated markers P16^INK4a^ and P21 in control and UVA-exposed HDFs. (**D**) Western blot analysis and quantification of MMP1 and Collagen-I expression. β-actin was used as the loading control. (**E**) RT-qPCR analysis of FUNDC1 mRNA expression in control and UVA-exposed HDFs. (**F**) Western blot analysis and quantification of FUNDC1 protein expression. β-actin was used as the loading control. (**G**) Representative Mtphagy Dye and Lyso Dye staining images and quantification of Mtphagy Dye fluorescence intensity. (**H**) Western blot analysis and quantification of LC3-II/LC3-I and P62 expression. β-actin was used as the loading control. (**I**) Representative DHE staining images and quantification of intracellular ROS fluorescence intensity. (**J**) ATP content in control and UVA-exposed HDFs. Data are presented as the mean ± SD from three independent biological replicates (*n* = 3). Statistical significance was determined by unpaired two-tailed Student’s *t*-test. Scale bars are indicated in the images. * *p* < 0.05, ** *p* < 0.01, *** *p* < 0.001.

To establish and validate a UVA-induced photoaging model in HDFs, cells were exposed to UVA and subsequently evaluated (Figure 1A). Compared with untreated control cells, UVA-exposed HDFs displayed enlarged and flattened morphology under phase-contrast microscopy (Supplementary Figure S1A). EdU incorporation was also reduced after UVA exposure, suggesting impaired proliferative capacity (Supplementary Figure S1B). To further characterize the photoaging phenotypes, SA-β-gal activity, senescence-associated gene expression, and skin aging-related proteins were examined. UVA-exposed HDFs showed an increased percentage of SA-β-gal-positive cells (Figure 1B), elevated mRNA levels of P16^INK4a^ and P21 (Figure 1C), increased MMP1 expression, and reduced Collagen-I expression (Figure 1D). These results confirmed the establishment of the UVA-induced HDF photoaging model.

FUNDC1 expression in UVA-induced photoaged HDFs was next assessed by RT-qPCR and Western blot analyses. Compared with the control group, FUNDC1 mRNA and protein levels were significantly reduced in UVA-exposed HDFs (Figure 1E,F). Since FUNDC1 is a mitophagy receptor involved in mitochondrial quality control, mitophagy-related activity was further examined. Mtphagy Dye staining showed weakened fluorescence in UVA-exposed HDFs compared with control cells (Figure 1G). In addition, the LC3-II/LC3-I ratio was decreased, whereas P62 expression was increased after UVA exposure (Figure 1H). These findings suggest diminished mitophagy-related activity and altered autophagy-associated signaling in UVA-induced photoaged HDFs. Consistently, UVA-exposed HDFs exhibited enhanced ROS levels, as indicated by stronger DHE fluorescence (Figure 1I), together with declining ATP content (Figure 1J). The above results indicate that UVA-induced photoaging is accompanied by impaired mitochondrial homeostasis in HDFs.

**Figure 2:**
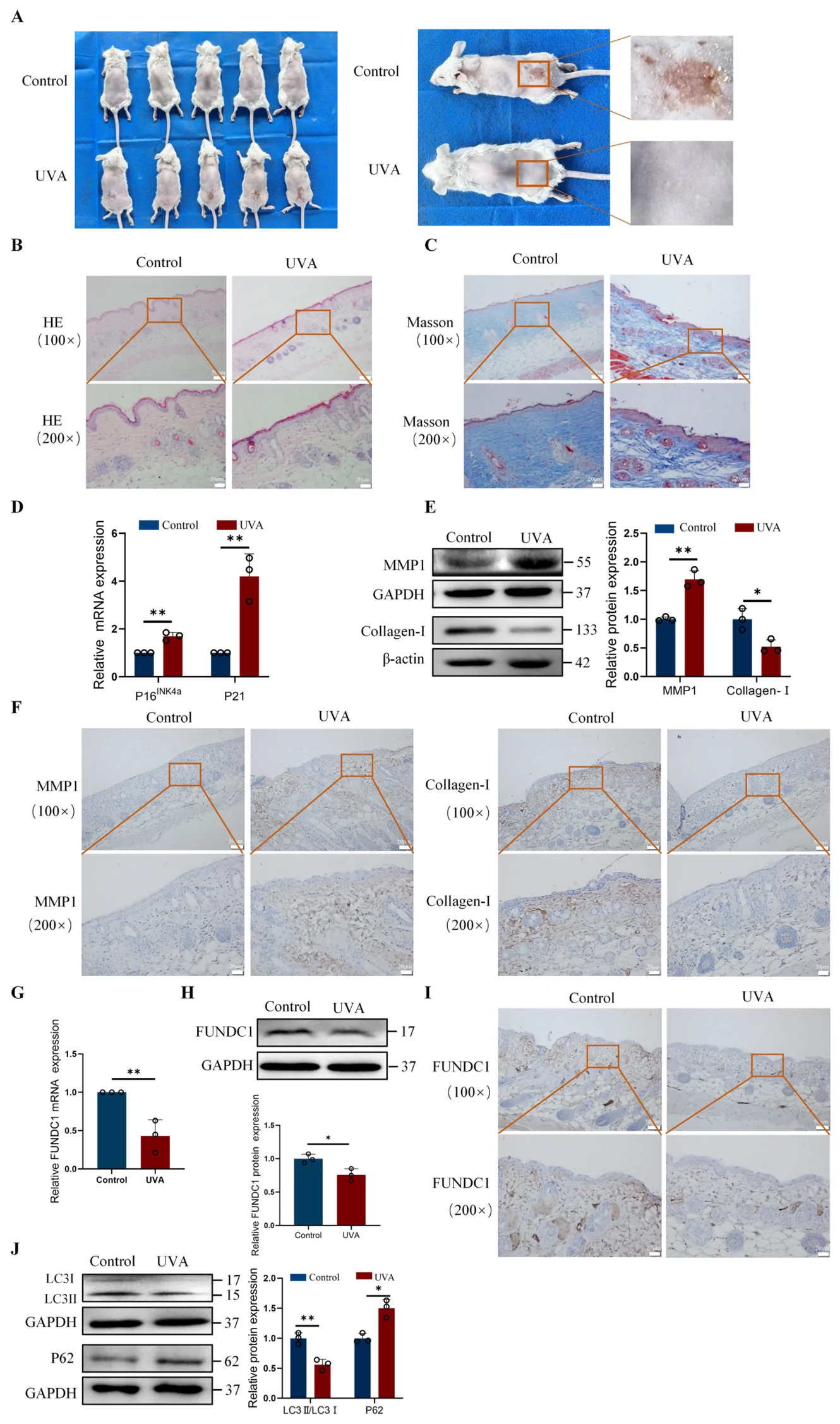
FUNDC1 downregulation and altered autophagy-associated signaling are observed in UVA-induced photoaged mouse skin. (**A**) Representative gross images of dorsal skin from control and UVA-irradiated mice, with enlarged views of the exposed skin area. (**B**) Hematoxylin and eosin staining of dorsal skin sections showing epidermal and dermal alterations after UVA exposure. (**C**) Masson’s trichrome staining showing collagen organization and deposition in control and UVA-irradiated mouse skin. (**D**) RT-qPCR analysis of P16^INK4a^ and P21 mRNA expression in mouse skin tissues. (**E**) Western blot analysis and quantification of MMP1 and Collagen-I expression. β-actin was used as the loading control. (**F**) Immunohistochemical staining of MMP1 and Collagen-I in mouse skin sections. (**G**) RT-qPCR analysis of FUNDC1 mRNA expression in mouse skin tissues. (**H**) Western blot analysis and quantification of FUNDC1 protein expression. GAPDH was used as the loading control. (**I**) Immunohistochemical staining of FUNDC1 in control and UVA-irradiated mouse skin. (**J**) Western blot analysis and quantification of LC3-II/LC3-I and P62 expression. GAPDH was used as the loading control. Gross morphological assessment, histological staining, and immunohistochemical staining were performed using five mice per group (*n* = 5), and representative images are shown in panels (**A**–**C**,**F**,**I**). RT-qPCR and Western blot analyses were performed using skin tissues from three independent mice per group (*n* = 3). Data are presented as the mean ± SD. Statistical significance was determined by unpaired two-tailed Student’s *t*-test. Scale bars are indicated in the images. * *p* < 0.05, ** *p* < 0.01.

To determine whether similar changes occurred in vivo, a UVA-induced mouse skin photoaging model was established. The dorsal skin of control mice appeared soft and smooth, whereas UVA-irradiated mice presented as rough and stiff skin with visible pigmentation and scabbing (Figure 2A). Hematoxylin and eosin staining and Masson’s trichrome staining showed that control skin had an intact epidermal structure, dense dermis, and well-organized collagen fibers. In contrast, UVA-exposed skin displayed epidermal thickening, dermal thinning, disorganized collagen fibers, and reduced collagen deposition (Figure 2B,C). The mRNA levels of P16^INK4a^ and P21 were elevated in UVA-irradiated mouse skin (Figure 2D). Immunohistochemistry and Western blot analyses further revealed increased MMP1 expression and decreased Collagen-I expression after UVA exposure (Figure 2E,F). These findings confirmed the establishment of the UVA-induced mouse skin photoaging model.

Consistent with the in vitro observations, FUNDC1 expression was remarkably reduced in photoaged mouse skin (Figure 2G–I). Moreover, UVA-exposed skin showed a decreased LC3-II/LC3-I ratio and increased P62 expression (Figure 2J), indicating altered autophagy-associated signaling in photoaged skin. Together, these in vitro and in vivo findings indicate that UVA-induced photoaging is accompanied by FUNDC1 downregulation, impaired mitochondrial homeostasis, and altered autophagy/mitophagy-related activity. Since FUNDC1 is closely associated with mitochondrial quality control, we next investigated whether altered FUNDC1 expression functionally influences UVA-induced photoaging in HDFs.

### 3.2. Fundc1 Attenuates UVA-Induced Photoaging and Mitochondrial Dysfunction in Hdfs

**Figure 3:**
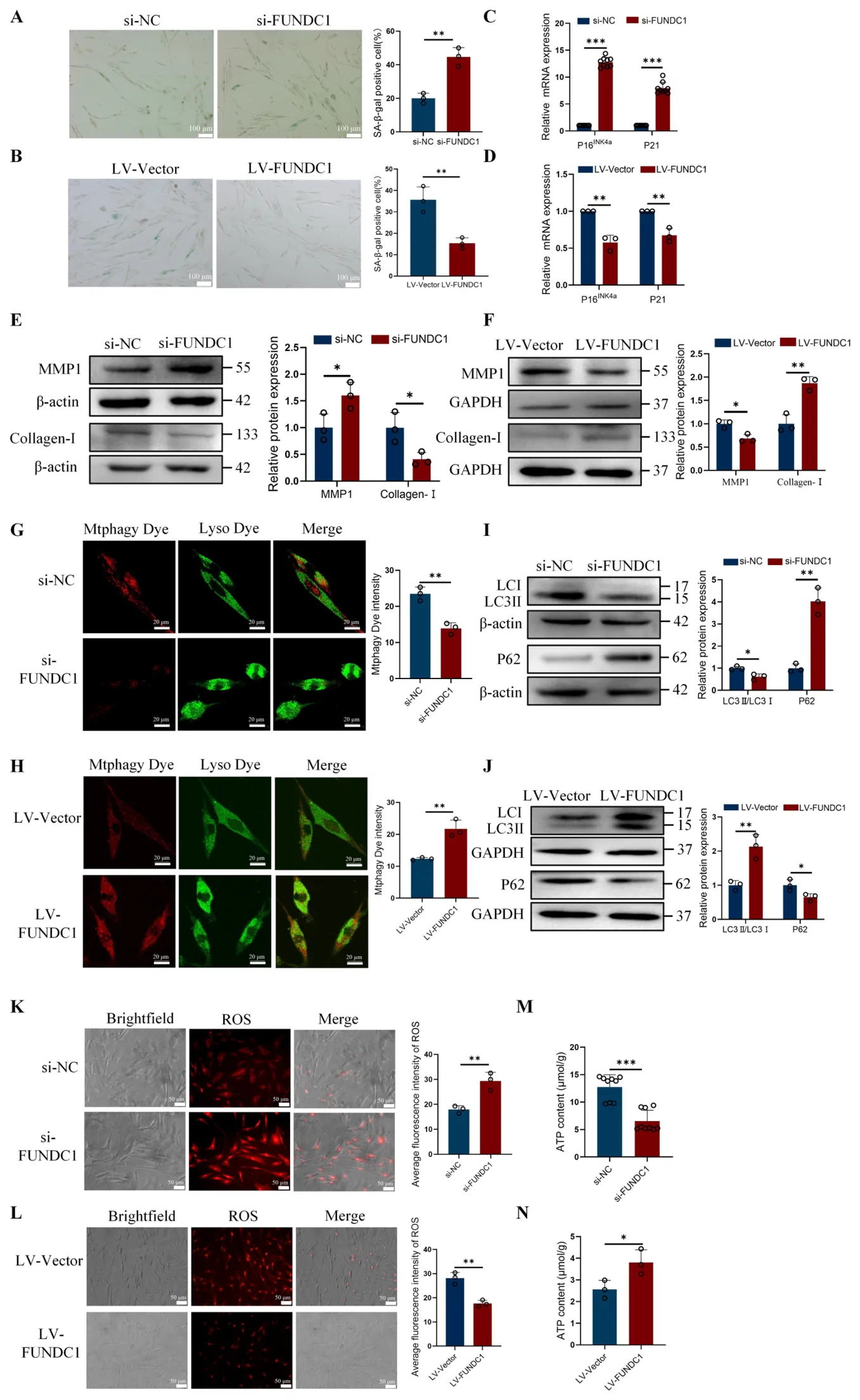
FUNDC1 attenuates photoaging-associated phenotypes and mitochondrial dysfunction in HDFs. (**A**,**B**) Representative SA-β-gal staining images and quantification of SA-β-gal-positive cells after FUNDC1 knockdown or FUNDC1 overexpression. (**C**,**D**) RT-qPCR analysis of P16^INK4a^ and P21 mRNA expression after FUNDC1 knockdown or FUNDC1 overexpression. (**E**,**F**) Western blot analysis and quantification of MMP1 and Collagen-I expression. β-actin or GAPDH was used as the loading control. (**G**,**H**) Representative Mtphagy Dye and Lyso Dye staining images and quantification of Mtphagy Dye fluorescence intensity after FUNDC1 knockdown or FUNDC1 overexpression. (**I**,**J**) Western blot analysis and quantification of LC3-II/LC3-I and P62 expression. β-actin or GAPDH was used as the loading control. (**K**,**L**) Representative DHE staining images and quantification of intracellular ROS fluorescence intensity. (**M**,**N**) ATP content after FUNDC1 knockdown or FUNDC1 overexpression. FUNDC1 knockdown was performed in HDFs, whereas FUNDC1 overexpression was performed in UVA-exposed HDFs to evaluate its protective effect under photoaging conditions. Data are presented as the mean ± SD from three independent biological replicates (*n* = 3). Statistical significance was determined by unpaired two-tailed Student’s *t*-test. Scale bars are indicated in the images. * *p* < 0.05, ** *p* < 0.01, *** *p* < 0.001.

Given the reduced FUNDC1 expression in UVA-induced photoaged HDFs, we next investigated whether FUNDC1 exerts a functional impact on HDF photoaging. FUNDC1 was either knocked down in HDFs or overexpressed in UVA-exposed HDFs. The knockdown and overexpression efficiencies were confirmed by RT-qPCR and Western blot analyses (Supplementary Figure S2A–E). We first examined senescence-associated phenotypes after FUNDC1 manipulation. FUNDC1 knockdown increased the percentage of SA-β-gal-positive cells, whereas FUNDC1 overexpression reduced UVA-induced SA-β-gal positivity (Figure 3A,B). Consistently, P16^INK4a^ and P21 mRNA levels were elevated after FUNDC1 knockdown but reduced by FUNDC1 overexpression in UVA-exposed HDFs (Figure 3C,D). Similar opposing effects were observed for skin aging-related proteins. FUNDC1 knockdown boosted MMP1 expression and weakened Collagen-I expression, whereas FUNDC1 overexpression decreased MMP1 and restored Collagen-I levels under UVA exposure (Figure 3E,F). These results indicate that FUNDC1 depletion promotes senescence-associated changes, whereas FUNDC1 overexpression attenuates UVA-induced photoaging phenotypes in HDFs. We further assessed mitophagy-related activity and mitochondrial homeostasis. FUNDC1 deficiency curtailed Mtphagy Dye fluorescence, whereas FUNDC1 overexpression enhanced Mtphagy Dye fluorescence in UVA-exposed HDFs (Figure 3G,H). Likewise, FUNDC1 knockdown decreased the LC3-II/LC3-I ratio and increased P62 expression, while FUNDC1 overexpression exerted the opposite effects under UVA exposure (Figure 3I,J). These findings suggest that FUNDC1 manipulation is associated with opposite changes in mitophagy-related activity and autophagy-associated signaling. In parallel, FUNDC1 knockdown fostered intracellular ROS accumulation and declined ATP content, whereas FUNDC1 replenishment mitigated ROS levels and restored ATP production in UVA-exposed HDFs (Figure 3K–N). Collectively, these findings indicate that FUNDC1 can modulate photoaging-associated phenotypes and mitochondrial homeostasis in HDFs, prompting us to further examine whether mitophagy-related processes are required for the protective effects associated with FUNDC1.

### 3.3. Mitophagy Contributes to Fundc1-Mediated Protection Against UVA-Induced Photoaging

**Figure 4:**
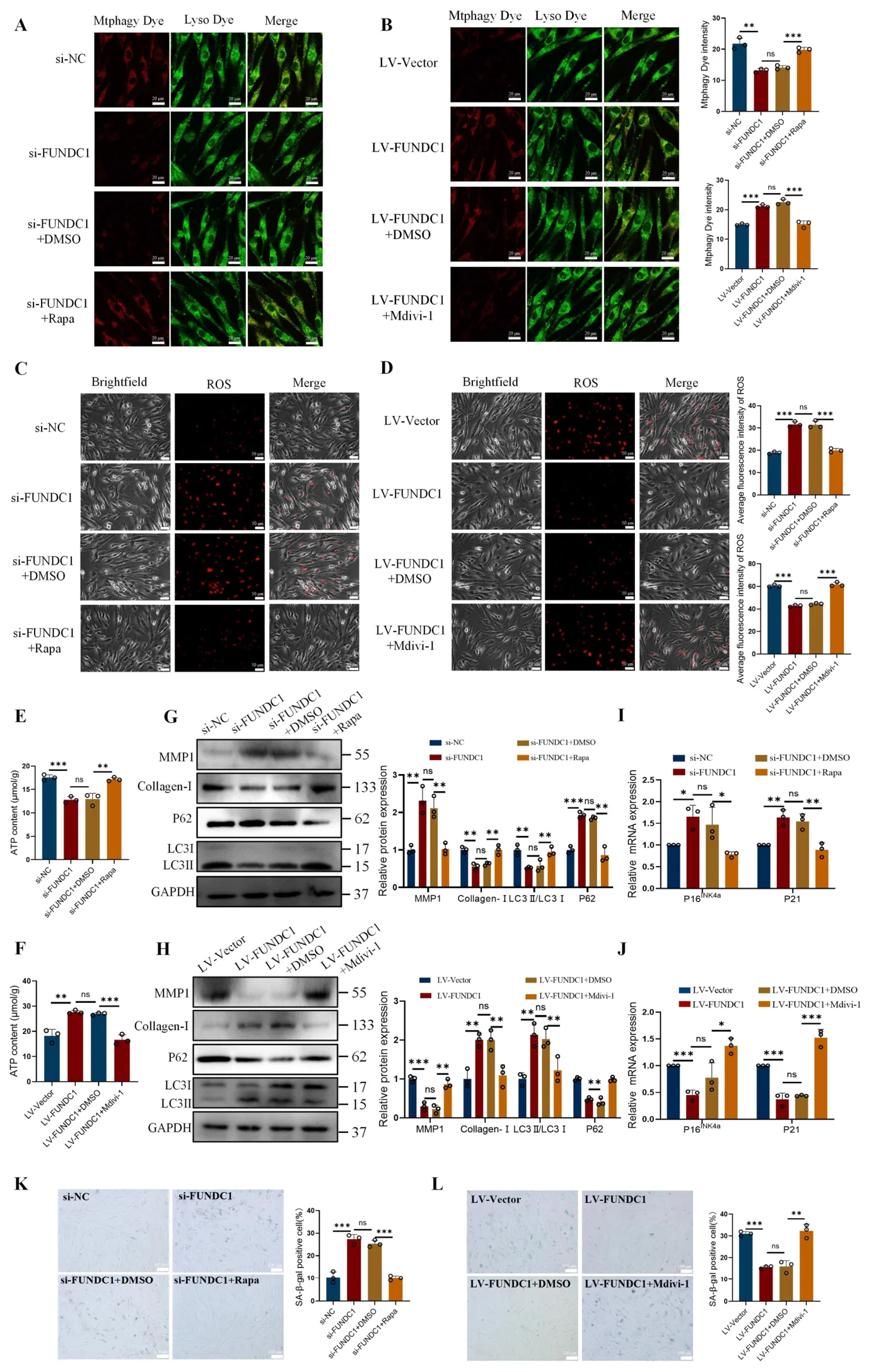
Mitophagy contributes to FUNDC1-mediated protection against HDF photoaging. (**A**) Representative Mtphagy Dye and Lyso Dye staining images and quantification of Mtphagy Dye fluorescence intensity in si-NC, si-FUNDC1, si-FUNDC1 + DMSO, and si-FUNDC1 + Rapa groups. (**B**) Representative Mtphagy Dye and Lyso Dye staining images and quantification of Mtphagy Dye fluorescence intensity in LV-Vector, LV-FUNDC1, LV-FUNDC1 + DMSO, and LV-FUNDC1 + Mdivi-1 groups. (**C**,**D**) Representative DHE staining images and quantification of intracellular ROS fluorescence intensity after Rapa or Mdivi-1 treatment. (**E**,**F**) ATP content after Rapa or Mdivi-1 treatment. (**G**,**H**) Western blot analysis and quantification of MMP1, Collagen-I, LC3-II/LC3-I, and P62 expression. GAPDH was used as the loading control. (**I**,**J**) RT-qPCR analysis of P16^INK4a^ and P21 mRNA expression. (**K**,**L**) Representative SA-β-gal staining images and quantification of SA-β-gal-positive cells. Rapa was used to activate autophagy/mitophagy-related activity under FUNDC1-deficient conditions, whereas Mdivi-1 was used to inhibit mitophagy-related processes in FUNDC1-overexpressing HDFs under UVA exposure. Data are presented as the mean ± SD from three independent biological replicates (*n* = 3). Statistical significance was determined by one-way ANOVA followed by Tukey’s multiple-comparisons test. Scale bars are indicated in the images. ns, not significant; * *p* < 0.05, ** *p* < 0.01, *** *p* < 0.001.

To further determine whether mitophagy-related processes participate in FUNDC1-mediated regulation of HDF photoaging, cells were treated with Rapa, a pharmacological activator of autophagy [20], or Mdivi-1, a commonly used pharmacological inhibitor of mitochondrial fission and mitophagy-related activity [21]. Because neither compound is entirely specific for mitophagy and both may affect additional cellular pathways, these pharmacological experiments were interpreted as supportive evidence for the involvement of mitophagy-related processes, rather than as definitive evidence that the observed effects are exclusively attributable to mitophagy. The effects of Rapa were first examined in FUNDC1-knockdown HDFs. Compared with the si-FUNDC1 + DMSO group, Rapa treatment restored Mtphagy Dye fluorescence (Figure 4A), increased the LC3-II/LC3-I ratio, and reduced P62 expression (Figure 4G). These results indicate that Rapa bolsters autophagy/mitophagy-related activity under FUNDC1-deficient conditions.

We next assessed mitochondrial functional changes in FUNDC1-knockdown HDFs. Compared with the si-FUNDC1 + DMSO group, Rapa treatment reduced intracellular ROS accumulation (Figure 4C) and boosted ATP content (Figure 4E). Meanwhile, Rapa downregulated P16^INK4a^ and P21 mRNA levels (Figure 4I), reduced MMP1 expression, restored Collagen-I expression (Figure 4G), and decreased the percentage of SA-β-gal-positive cells (Figure 4K). These findings suggest that activation of autophagy by Rapa attenuates mitochondrial dysfunction and photoaging-like changes caused by FUNDC1 knockdown.

We then examined whether inhibition of mitophagy affects the protective effects of FUNDC1 overexpression under UVA exposure. In UVA-exposed HDFs overexpressing FUNDC1, Mdivi-1 treatment alleviated Mtphagy Dye fluorescence compared with the LV-FUNDC1 + DMSO group (Figure 4B), accompanied by a decreased LC3-II/LC3-I ratio and increased P62 expression (Figure 4H). Mdivi-1 also enhanced ROS accumulation (Figure 4D) and reduced ATP content (Figure 4F), indicating that inhibition of mitophagy weakened the improvement of mitochondrial function associated with FUNDC1 repletion. Consistently, Mdivi-1 partially reversed the protective effects of FUNDC1 overexpression on UVA-induced photoaging phenotypes. Compared with the LV-FUNDC1 + DMSO group, the LV-FUNDC1 + Mdivi-1 group showed elevated P16^INK4a^ and P21 mRNA levels (Figure 4J), increased MMP1 expression, reduced Collagen-I expression (Figure 4H), and a higher percentage of SA-β-gal-positive cells (Figure 4L). Together, these data indicate that Rapa partially rescues the detrimental effects caused by FUNDC1 knockdown, whereas Mdivi-1 weakens the protective effects of FUNDC1 overexpression. Thus, mitophagy participates in FUNDC1-mediated protection against HDF photoaging.

**Figure 5:**
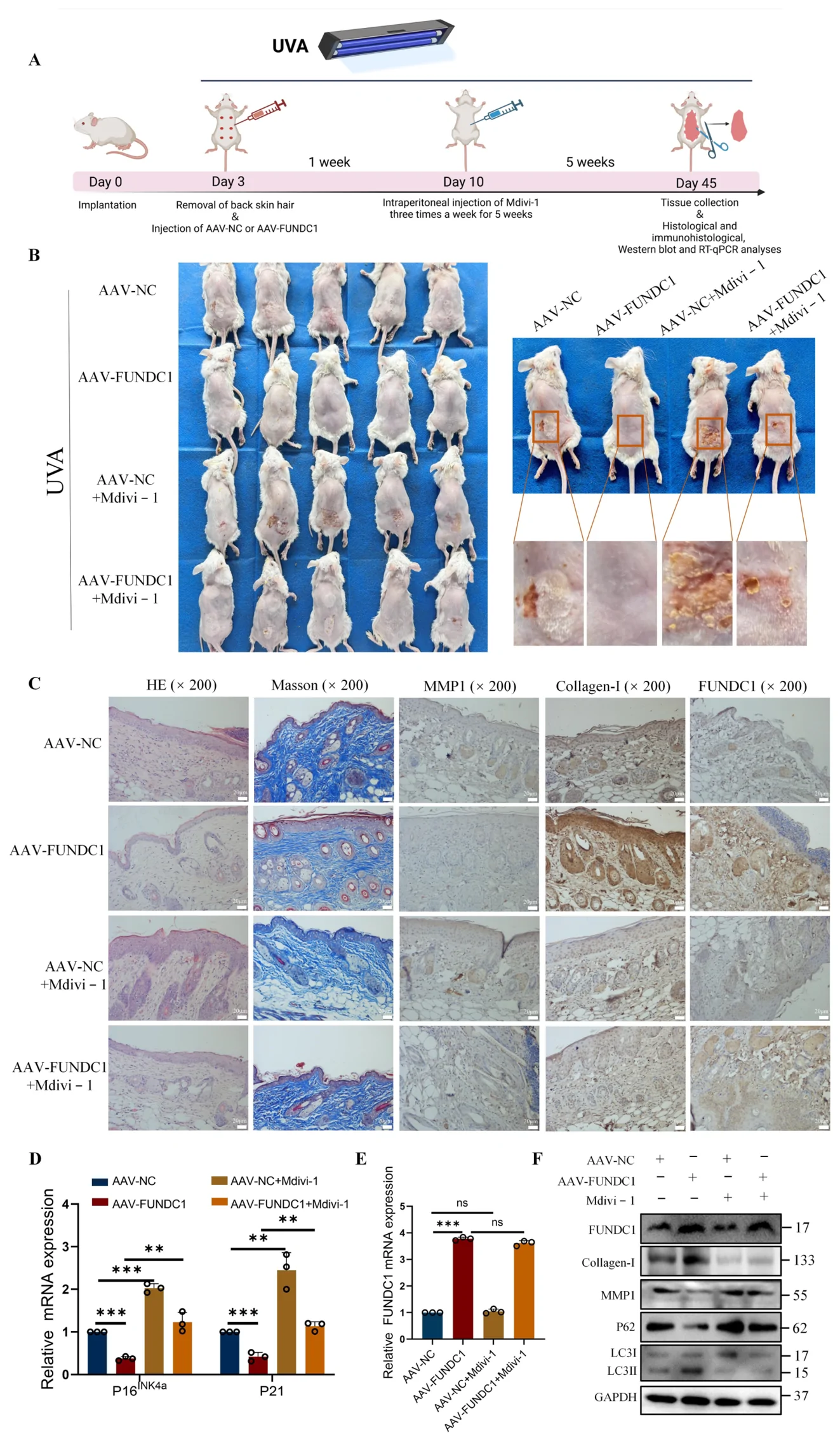
Mdivi-1 attenuates the protective effects of AAV-FUNDC1 against UVA-induced skin photoaging in vivo. (**A**) Schematic illustration of the in vivo experimental design. Mice were treated with AAV-NC or AAV-FUNDC1, followed by Mdivi-1 administration under UVA-induced photoaging conditions. (**B**) Representative gross images of dorsal skin and enlarged views of the treated skin area from AAV-NC, AAV-FUNDC1, AAV-NC + Mdivi-1, and AAV-FUNDC1 + Mdivi-1 groups. (**C**) Hematoxylin and eosin staining, Masson’s trichrome staining, and immunohistochemical staining of MMP1, Collagen-I and FUNDC1 in mouse skin sections. (**D**) RT-qPCR analysis of P16^INK4a^ and P21 mRNA expression in mouse skin tissues. (**E**) RT-qPCR analysis of FUNDC1 mRNA expression. (**F**) Western blot analysis of FUNDC1, Collagen-I, MMP1, LC3-II/LC3-I, and P62 expression. GAPDH was used as the loading control. Gross morphological assessment, histological staining, and immunohistochemical staining were performed using five mice per group (*n* = 5), and representative images are shown in panels (**B**,**C**). RT-qPCR and Western blot analyses were performed using skin tissues from three independent mice per group (*n* = 3). Data are presented as the mean ± SD. Statistical significance was determined by two-way ANOVA followed by Tukey’s multiple-comparisons test. Scale bars are indicated in the images. ns, not significant, ** *p* < 0.01, *** *p* < 0.001.

To further validate this relationship in vivo, AAV-mediated FUNDC1 overexpression was combined with Mdivi-1 treatment in a UVA-induced mouse skin photoaging model (Figure 5A). Compared with AAV-NC mice, AAV-FUNDC1-treated mice showed visibly smoother dorsal skin, reduced epidermal thickening, and more organized dermal collagen fibers after UVA exposure (Figure 5B,C). In contrast, Mdivi-1 treatment aggravated photoaging-like skin changes, including rougher skin appearance, increased epidermal thickness, and disrupted collagen organization. Notably, co-administration of Mdivi-1 partially attenuated the protective effects of AAV-FUNDC1, as shown by increased skin roughness, epidermal thickening, and collagen disorganization compared with the AAV-FUNDC1 group (Figure 5B,C). Consistent with the histological observations, AAV-FUNDC1 reduced the mRNA levels of P16^INK4a^ and P21 in photoaged mouse skin, whereas Mdivi-1 treatment increased their expression. The inhibitory effects of AAV-FUNDC1 on these senescence markers were partially reversed by Mdivi-1 co-treatment (Figure 5D). AAV-FUNDC1 also upregulated FUNDC1 expression in mouse skin, and this expression remained elevated in the AAV-FUNDC1 + Mdivi-1 group (Figure 5E,F). Western blot analysis further showed that AAV-FUNDC1 increased Collagen-I expression, reduced MMP1 expression, incremented the LC3-II/LC3-I ratio, and diminished P62 expression. However, these effects were partially antagonized by Mdivi-1 treatment (Figure 5F). Collectively, these in vitro and in vivo findings indicate that mitophagy participates in the protective effects of FUNDC1 against UVA-induced photoaging.

### 3.4. Mir-137-3p Negatively Regulates Fundc1 Expression During Photoaging-Associated Mitochondrial Dysfunction in Hdfs

**Figure 6:**
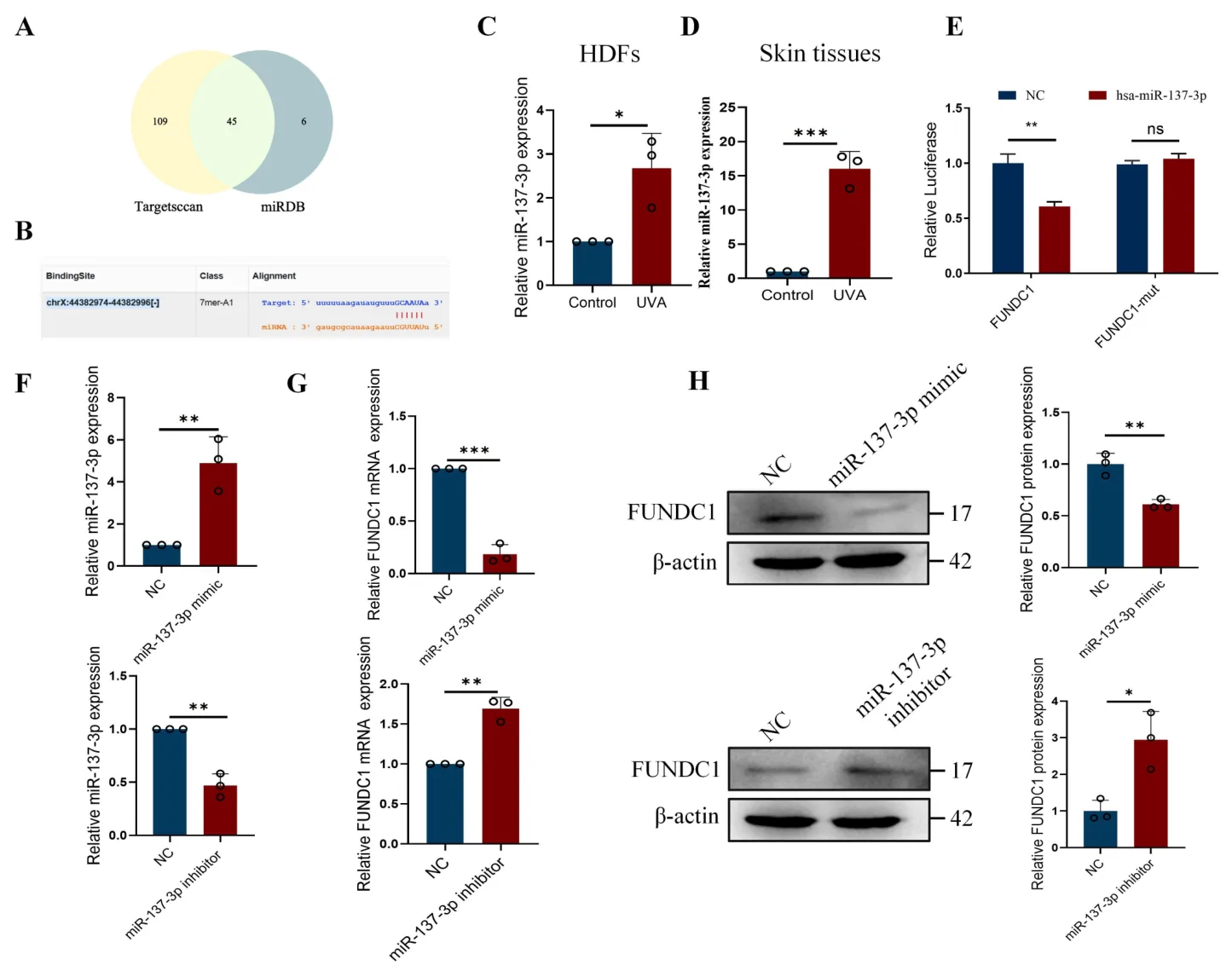
MiR-137-3p directly targets the FUNDC1 3′UTR and negatively regulates FUNDC1 expression in HDFs. (**A**) Venn diagram showing overlapping candidate miRNAs predicted to target the FUNDC1 3′UTR by TargetScan and miRDB. (**B**) Predicted binding site of miR-137-3p within the FUNDC1 3′UTR. (**C**) RT-qPCR analysis of miR-137-3p expression in control and UVA-exposed HDFs. (**D**) RT-qPCR analysis of miR-137-3p expression in control and UVA-irradiated mouse skin tissues. (**E**) Dual-luciferase reporter assay using wild-type or mutant FUNDC1 3′UTR reporter constructs after miR-137-3p mimic transfection. (**F**) RT-qPCR validation of miR-137-3p mimic and miR-137-3p inhibitor transfection efficiency. (**G**) RT-qPCR analysis of FUNDC1 mRNA expression after miR-137-3p mimic or inhibitor transfection. (**H**) Western blot analysis and quantification of FUNDC1 protein expression after miR-137-3p mimic or inhibitor transfection. β-actin was used as the loading control. Data are presented as the mean ± SD from three independent biological replicates (n = 3). Statistical significance was determined by unpaired two-tailed Student’s *t*-test for (**C**,**D**), and (**F**–**H**). For (**E**), data were analyzed by two-way ANOVA followed by Tukey’s multiple-comparisons test. ns, not significant; * *p* < 0.05, ** *p* < 0.01, *** *p* < 0.001.

Given the widespread involvement of microRNAs (miRNAs) in post-transcriptional gene regulation and cellular senescence, we next explored whether miRNAs contribute to FUNDC1 downregulation during UVA-induced HDF photoaging. FUNDC1 was used as the target gene for miRNA prediction using Targetscan and miRDB. Intersection analysis of the two prediction datasets identified 45 overlapping candidate miRNAs potentially targeting the FUNDC1 3′UTR (Figure 6A). Among these candidates, miR-137-3p was prioritized for further validation because previous studies have reported that miR-137 regulates mitophagy by targeting FUNDC1 and NIX under hypoxic conditions [13] (Figure 6B). However, it remains unclear whether miR-137-3p is altered during UVA-induced skin photoaging and whether the miR-137-3p/FUNDC1 regulatory relationship contributes to impaired mitophagy and photoaging-associated phenotypes. Therefore, miR-137-3p was selected for further investigation in the present study. We first examined miR-137-3p expression in UVA-induced photoaging models. RT-qPCR analysis showed that miR-137-3p was significantly upregulated in UVA-exposed HDFs compared with control HDFs (Figure 6C). Consistently, miR-137-3p expression was also increased in UVA-exposed mouse skin compared with control mouse skin (Figure 6D). These findings indicate that miR-137-3p is upregulated during UVA-induced photoaging both in vitro and in vivo. To validate the predicted interaction with the FUNDC1 3′UTR, we analyzed the predicted binding site and performed a dual-luciferase reporter assay. The predicted miR-137-3p binding sequence was located within the FUNDC1 3′UTR (Figure 6B). The miR-137-3p mimic significantly reduced the luciferase activity of the wild-type FUNDC1 3′UTR reporter, whereas mutation of the predicted binding site abolished this inhibitory effect (Figure 6E). These results support that miR-137-3p regulates FUNDC1 through the predicted 3′UTR binding site. We next examined whether miR-137-3p regulates endogenous FUNDC1 expression in HDFs. Transfection with the miR-137-3p mimic markedly increased miR-137-3p expression, whereas transfection with the miR-137-3p inhibitor reduced miR-137-3p expression (Figure 6F). Consistently, the miR-137-3p mimic decreased FUNDC1 mRNA and protein levels, while miR-137-3p inhibition had the opposite effect (Figure 6G,H). Altogether, these findings support miR-137-3p as an upstream negative regulator of FUNDC1 expression in HDFs.

Having confirmed that miR-137-3p is upregulated during UVA-induced photoaging and negatively regulates FUNDC1 expression, we next investigated the functional relevance of miR-137-3p in photoaging-associated cellular phenotypes and mitochondrial homeostasis. For gain-of-function analysis, the miR-137-3p mimic was transfected into HDFs to simulate the elevation in miR-137-3p expression observed during UVA-induced photoaging. For loss-of-function analysis, the miR-137-3p inhibitor was transfected into UVA-exposed HDFs to suppress the elevated endogenous miR-137-3p levels under photoaging conditions.

**Figure 7:**
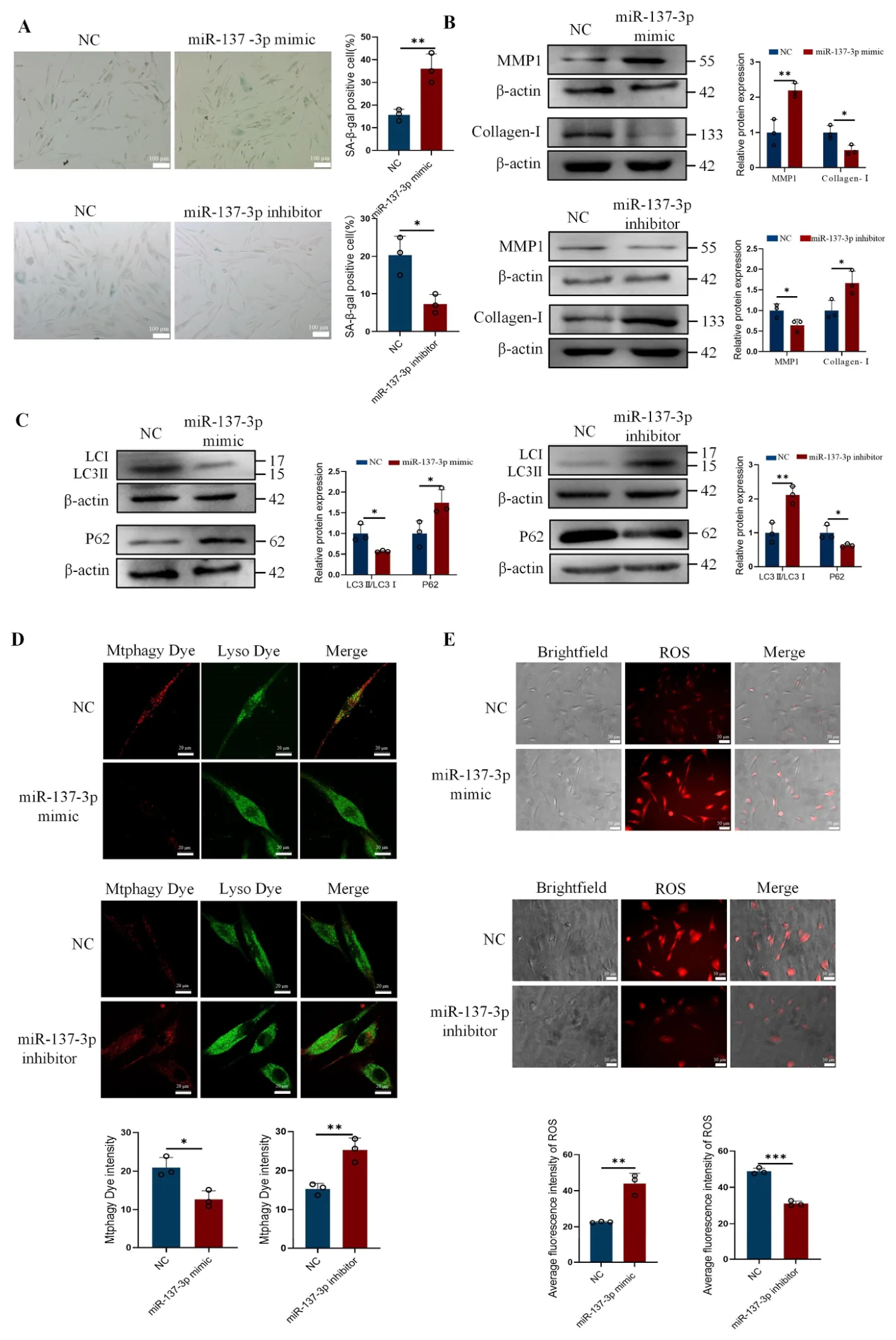
MiR-137-3p promotes photoaging-associated phenotypes and mitochondrial dysfunction in HDFs. (**A**) Representative SA-β-gal staining images and quantification of SA-β-gal-positive cells after miR-137-3p mimic or inhibitor transfection. (**B**) Western blot analysis and quantification of MMP1 and Collagen-I expression after miR-137-3p mimic or inhibitor transfection. β-actin was used as the loading control. (**C**) Western blot analysis and quantification of LC3-II/LC3-I and P62 expression. β-actin was used as the loading control. (**D**) Representative Mtphagy Dye and Lyso Dye staining images and quantification of Mtphagy Dye fluorescence intensity. (**E**) Representative DHE staining images and quantification of intracellular ROS fluorescence intensity. For gain-of-function analysis, HDFs were transfected with miR-137-3p mimic. For loss-of-function analysis, UVA-exposed HDFs were transfected with miR-137-3p inhibitor. Data are presented as the mean ± SD from three independent biological replicates (n = 3). Statistical significance was determined by unpaired two-tailed Student’s *t*-test. Scale bars are indicated in the images. * *p* < 0.05, ** *p* < 0.01, *** *p* < 0.001.

Transfection with the miR-137-3p mimic increased the proportion of SA-β-gal-positive cells compared with the negative control group, whereas inhibition of miR-137-3p reduced SA-β-gal positivity in UVA-exposed HDFs (Figure 7A). Furthermore, the miR-137-3p mimic strengthened MMP1 expression and weakened Collagen-I expression, while miR-137-3p inhibition in UVA-exposed HDFs decreased MMP1 expression and restored Collagen-I expression (Figure 7B). The mRNA levels of P16^INK4a^ and P21 showed similar changes after miR-137-3p mimic or inhibitor transfection (Supplementary Figure S3A). These findings suggest that miR-137-3p replenishment triggers photoaging-associated phenotypes in HDFs, whereas inhibition of miR-137-3p attenuates these changes under UVA-induced photoaging conditions. We further assessed mitophagy-related activity and mitochondrial function after miR-137-3p manipulation. The miR-137-3p mimic decreased the LC3-II/LC3-I ratio, increased P62 expression, and reduced Mtphagy Dye fluorescence, whereas miR-137-3p inhibition in UVA-exposed HDFs increased the LC3-II/LC3-I ratio, reduced P62 expression, and enhanced Mtphagy Dye fluorescence (Figure 7C,D). In addition, the miR-137-3p mimic led to excessive ROS accumulation and decreased ATP content, while miR-137-3p inhibition reduced ROS accumulation and restored ATP production in UVA-exposed HDFs (Figure 7E and Figure S3B). Together, these results support that elevated miR-137-3p is associated with reduced mitophagy-related activity, impaired mitochondrial function, and photoaging-associated phenotypes in HDFs.

To further explore the functional relationship between miR-137-3p and FUNDC1, we performed an additional co-transfection analysis using si-FUNDC1 and the miR-137-3p inhibitor. si-FUNDC1 reduced FUNDC1 mRNA and protein expression, whereas co-transfection with the miR-137-3p inhibitor partially restored FUNDC1 expression compared with the si-FUNDC1 + NC inhibitor group (Supplementary Figure S4A,B). Because si-FUNDC1 targets the coding sequence of FUNDC1, whereas miR-137-3p acts through the FUNDC1 3′UTR, these two interventions target distinct regions of the FUNDC1 transcript. The partial restoration may therefore reflect relief of miR-137-3p-mediated repression of the residual FUNDC1 transcripts remaining after siRNA-mediated knockdown. Consistently, miR-137-3p inhibition partially counteracted several cellular changes associated with FUNDC1 knockdown, including increased SA-β-gal positivity, elevated P16^INK4a^ and P21 mRNA levels, increased MMP1 expression, and reduced Collagen-I expression (Supplementary Figure S4C–E). In addition, miR-137-3p inhibition partially attenuated the decrease in mitophagy-related activity and mitochondrial function caused by FUNDC1 knockdown, as reflected by partial recovery of LC3-II/LC3-I and P62 expression, increased Mtphagy Dye fluorescence, reduced ROS accumulation, and improved ATP production (Supplementary Figure S4F–I). These findings further support a functional association between miR-137-3p and FUNDC1 in regulating photoaging-associated cellular changes in HDFs. Overall, these results suggest that miR-137-3p is upregulated during UVA-induced photoaging, negatively regulates FUNDC1 expression, and is functionally associated with impaired mitophagy-related activity, mitochondrial dysfunction, and photoaging-associated cellular changes in HDFs.

### 3.5. Fundc1 Regulates P53 Ubiquitination and Stability in a Baz1b-Associated Manner

**Figure 8:**
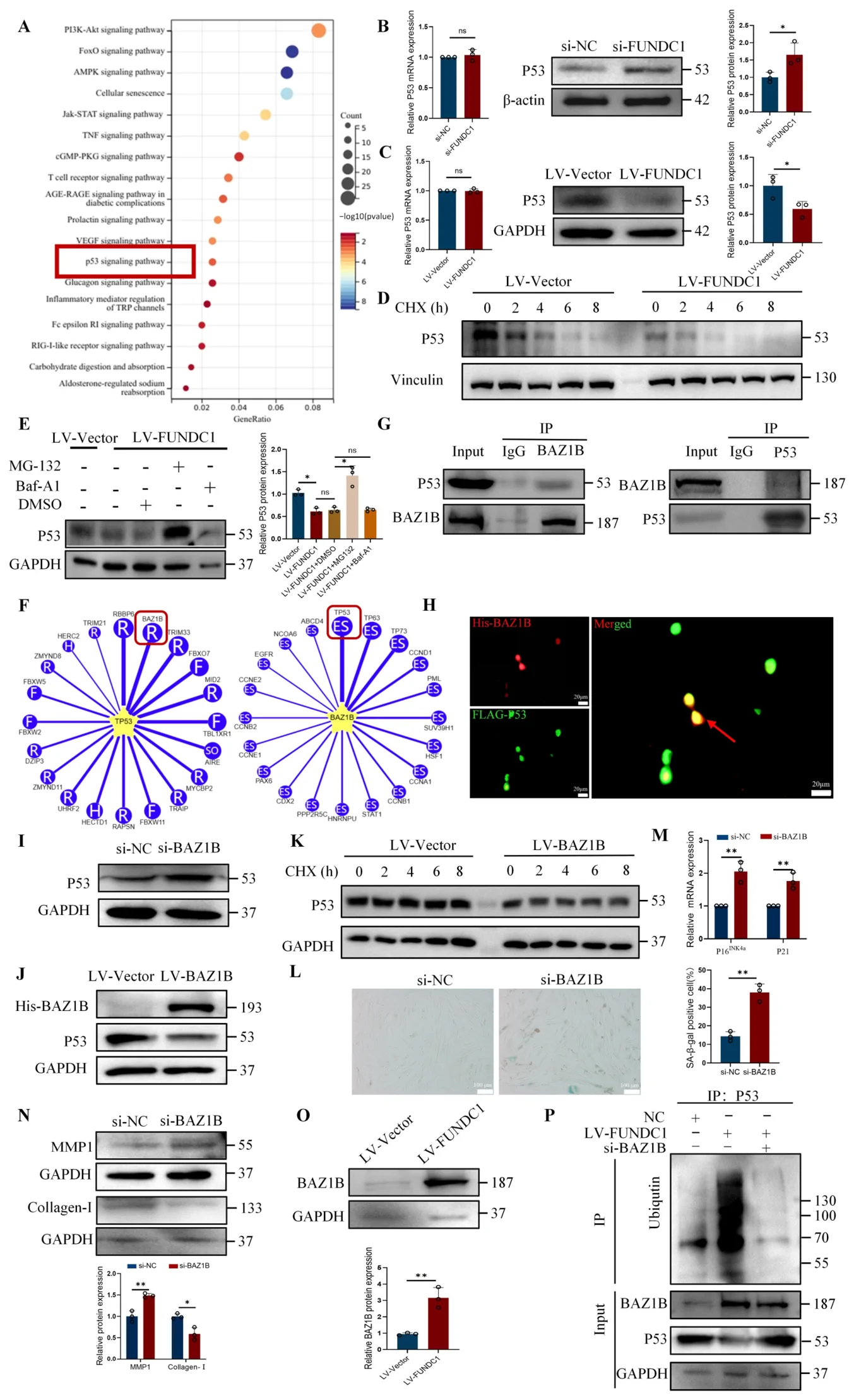
FUNDC1 is associated with BAZ1B-related regulation of P53 ubiquitination and protein stability. (**A**) KEGG pathway enrichment analysis of differentially expressed autophagy-related genes from the GEO dataset GSE119009, highlighting enrichment of the P53 signaling pathway. (**B**) RT-qPCR analysis of P53 mRNA expression and Western blot analysis of P53 protein expression after FUNDC1 knockdown. β-actin was used as the loading control. (**C**) RT-qPCR analysis of P53 mRNA expression and Western blot analysis of P53 protein expression after FUNDC1 overexpression. GAPDH was used as the loading control. (**D**) CHX chase analysis of P53 protein turnover in LV-Vector and LV-FUNDC1 HDFs at the indicated time points. P53 protein levels were normalized to GAPDH and further normalized to the corresponding 0 h level. (**E**) Western blot analysis of P53 expression in LV-Vector and LV-FUNDC1 HDFs after treatment with DMSO, MG132, or Baf-A1. GAPDH was used as the loading control. (**F**) UbiBrowser-based prediction of candidate ubiquitination regulators associated with P53, with BAZ1B identified among the top-ranked candidates. (**G**) Co-immunoprecipitation analysis showing an association between endogenous BAZ1B and P53. (**H**) Representative fluorescence colocalization images of His-BAZ1B and FLAG-P53 in cells. Arrows indicate colocalized signals. (**I**,**J**) Western blot analysis of P53 protein expression after BAZ1B knockdown or BAZ1B overexpression. GAPDH was used as the loading control. (**K**) CHX chase analysis of P53 protein turnover in LV-Vector and LV-BAZ1B cells at the indicated time points. (**L**) Representative SA-β-gal staining images and quantification of SA-β-gal-positive cells after BAZ1B knockdown. (**M**) RT-qPCR analysis of P16^INK4a^ and P21 mRNA expression after BAZ1B knockdown. (**N**) Western blot analysis and quantification of MMP1 and Collagen-I expression after BAZ1B knockdown. GAPDH was used as the loading control. (**O**) Western blot analysis and quantification of BAZ1B protein expression after FUNDC1 overexpression. GAPDH was used as the loading control. (**P**) Immunoprecipitation analysis of P53 ubiquitination after FUNDC1 overexpression with or without BAZ1B knockdown. P53 was immunoprecipitated, and ubiquitinated P53 was detected by immunoblotting with an anti-ubiquitin antibody. Data are presented as the mean ± SD from three independent biological replicates (n = 3). Comparisons between two independent groups were performed using unpaired two-tailed Student’s *t*-tests. Data in (**E**) were analyzed using one-way ANOVA followed by Tukey’s multiple-comparisons test. Scale bars are indicated in the images. ns, not significant, * *p* < 0.05, ** *p* < 0.01.

It has been demonstrated that FUNDC1 can modulate mitophagy-related mitochondrial homeostasis during HDF photoaging, we next explored additional downstream pathways that might be associated with FUNDC1-mediated regulation of photoaging-associated cellular alterations. To this end, differentially expressed genes (DEGs) were extracted from the GEO dataset GSE119009 and intersected with an autophagy-related gene set. KEGG pathway enrichment analysis of the intersected genes revealed enrichment of multiple signaling pathways, including the P53 signaling pathway (Figure 8A). Given the well-established involvement of P53 signaling in cellular senescence, the P53 pathway was selected for further investigation as a candidate pathway associated with FUNDC1-related regulation of HDF photoaging. We first examined whether FUNDC1 affects P53 expression. FUNDC1 knockdown or overexpression did not markedly alter P53 mRNA levels, whereas P53 protein expression was changed after FUNDC1 manipulation. FUNDC1 knockdown upregulated P53 protein levels, while FUNDC1 overexpression downregulated P53 protein expression (Figure 8B,C). CHX chase analysis further revealed that P53 protein declined more rapidly in FUNDC1-overexpressing cells than in control cells after normalization to the corresponding 0 h level, suggesting that FUNDC1 overexpression is related to increased P53 protein turnover (Figure 8D). Since P53 protein levels seemed to be regulated post-transcriptionally, we further examined whether protein degradation pathways were involved. Treatment with the proteasome inhibitor MG132, but not the lysosomal inhibitor Baf-A1, attenuated the reduction in P53 protein levels caused by FUNDC1 overexpression (Figure 8E). These findings suggest that FUNDC1 is implicated in proteasome-dependent regulation of P53 protein stability.

To identify potential ubiquitination regulators involved in P53 turnover, candidate E3 ubiquitin ligases associated with P53 were predicted using the UbiBrowser database. Among the top-ranked candidates, BAZ1B exhibited a high predicted interaction score with P53 and had not been extensively characterized in this context (Figure 8F). Co-immunoprecipitation assays supported an association between BAZ1B and P53, and fluorescence colocalization analysis showed partial colocalization of BAZ1B with P53 in cells (Figure 8G,H). Functional analysis further displayed that BAZ1B silencing (Supplementary Figure S5A,B) increased P53 protein levels, whereas BAZ1B sufficiency reduced P53 protein expression (Figure 8I,J). CHX chase analysis showed that P53 protein declined more rapidly in BAZ1B-overexpressing cells than in control cells, supporting a role for BAZ1B in regulating P53 protein turnover (Figure 8K). These results suggest that BAZ1B participates in the regulation of P53 protein stability.

Moreover, we examined whether BAZ1B expression was functionally associated with photoaging-related phenotypes. BAZ1B knockdown increased the proportion of SA-β-gal-positive cells, elevated P16^INK4a^ and P21 mRNA levels, enhanced MMP1 expression, and reduced Collagen-I expression in HDFs (Figure 8L–N). Consistent with the results of the cell experiments, BAZ1B expression was downregulated in UVA-induced photoaged skin, whereas P53 expression was upregulated and P53 ubiquitination levels were abolished (Supplementary Figure S5C–E). These findings suggest that reduced BAZ1B expression may be correlated with P53 accumulation and photoaging-associated cellular changes.

We further investigated whether BAZ1B is involved in FUNDC1-associated regulation of P53 ubiquitination. We found that FUNDC1 overexpression was associated with increased BAZ1B protein expression, suggesting that BAZ1B may participate in FUNDC1-associated regulation of P53 stability (Figure 8O). However, this result does not define the molecular mechanism by which FUNDC1 influences BAZ1B abundance. Co-immunoprecipitation analysis showed that FUNDC1 overexpression enhanced P53 ubiquitination, whereas BAZ1B knockdown attenuated this effect (Figure 8P). Together, these results suggest that BAZ1B is involved in FUNDC1-associated regulation of P53 ubiquitination and protein stability.

### 3.6. P53 K292 Contributes to Baz1b-Associated P53 Ubiquitination

**Figure 9:**
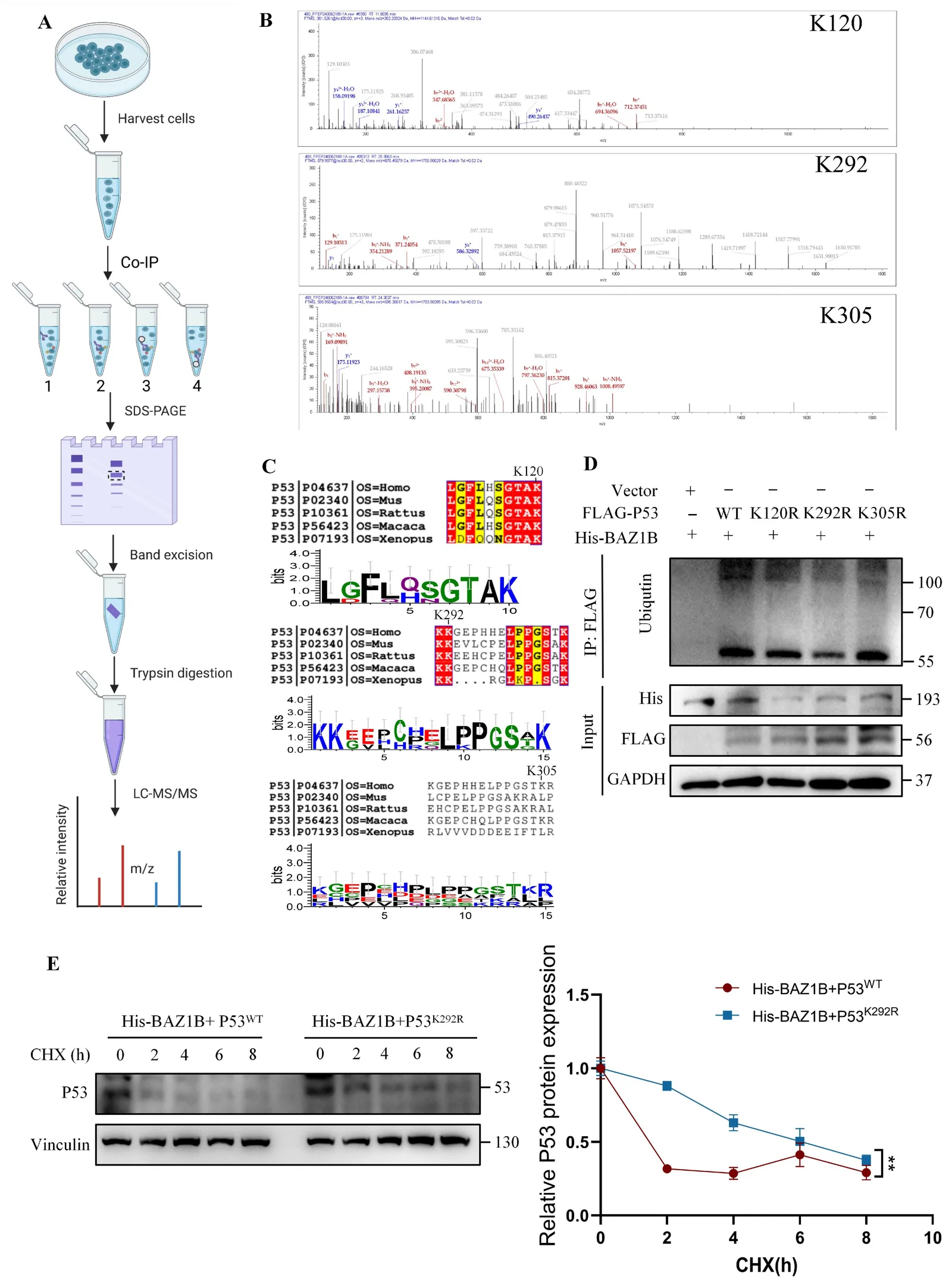
P53 K292 serves as a major ubiquitination site associated with BAZ1B-related regulation of P53 stability. (**A**) Schematic workflow for LC-MS/MS-based identification of potential ubiquitination sites on P53. (**B**) Representative LC-MS/MS spectra identifying K120, K292 and K305 as candidate ubiquitination sites on P53. (**C**) Cross-species sequence alignment and conservation logo analysis of the identified candidate ubiquitination sites. (**D**) FLAG immunoprecipitation followed by ubiquitin immunoblotting in HEK293 cells co-transfected with His-BAZ1B and FLAG-tagged P53WT, P53K120R, P53K292R, or P53K305R constructs. His-BAZ1B, FLAG-P53, and GAPDH were detected in input lysates. (**E**) CHX chase analysis comparing the turnover of P53WT and P53K292R in the presence of His-BAZ1B at the indicated time points. P53 protein levels were normalized to GAPDH and further normalized to the corresponding 0 h level. Data are presented as the mean ± SD from three independent biological replicates (n = 3). Data in panel (**E**) were analyzed using two-way ANOVA followed by Tukey’s multiple-comparisons test. ** *p* < 0.01.

To further explore potential ubiquitination sites on P53, LC-MS/MS analysis was performed and identified three candidate ubiquitination sites: K120, K292, and K305 (Figure 9A,B). As P53 is highly conserved across species, sequence alignment and conservation analysis were then performed. Among the identified candidate sites, K120 and K292 showed relatively high conservation across species (Figure 9C). To evaluate the functional relevance of these lysine residues, P53 mutants carrying lysine-to-arginine substitutions at K120, K292, or K305 were generated and co-transfected with BAZ1B into HEK293 cells. FLAG immunoprecipitation followed by ubiquitin immunoblotting indicated that the K292R mutation attenuated BAZ1B-associated P53 ubiquitination more evidently than the K120R or K305R mutations (Figure 9D). Furthermore, after normalization to the corresponding 0 h level, CHX chase analysis presented that the P53K292R mutant declined more slowly than P53WT in the presence of BAZ1B, implying that mutation of K292 may reduce BAZ1B-associated P53 turnover (Figure 9E). Overall, these findings suggest that K292 is a major site involved in BAZ1B-associated regulation of P53 ubiquitination and stability.

### 3.7. Baz1b Knockdown Attenuates Fundc1-Associated Protection Against UVA-Induced Skin Photoaging In Vivo

**Figure 10:**
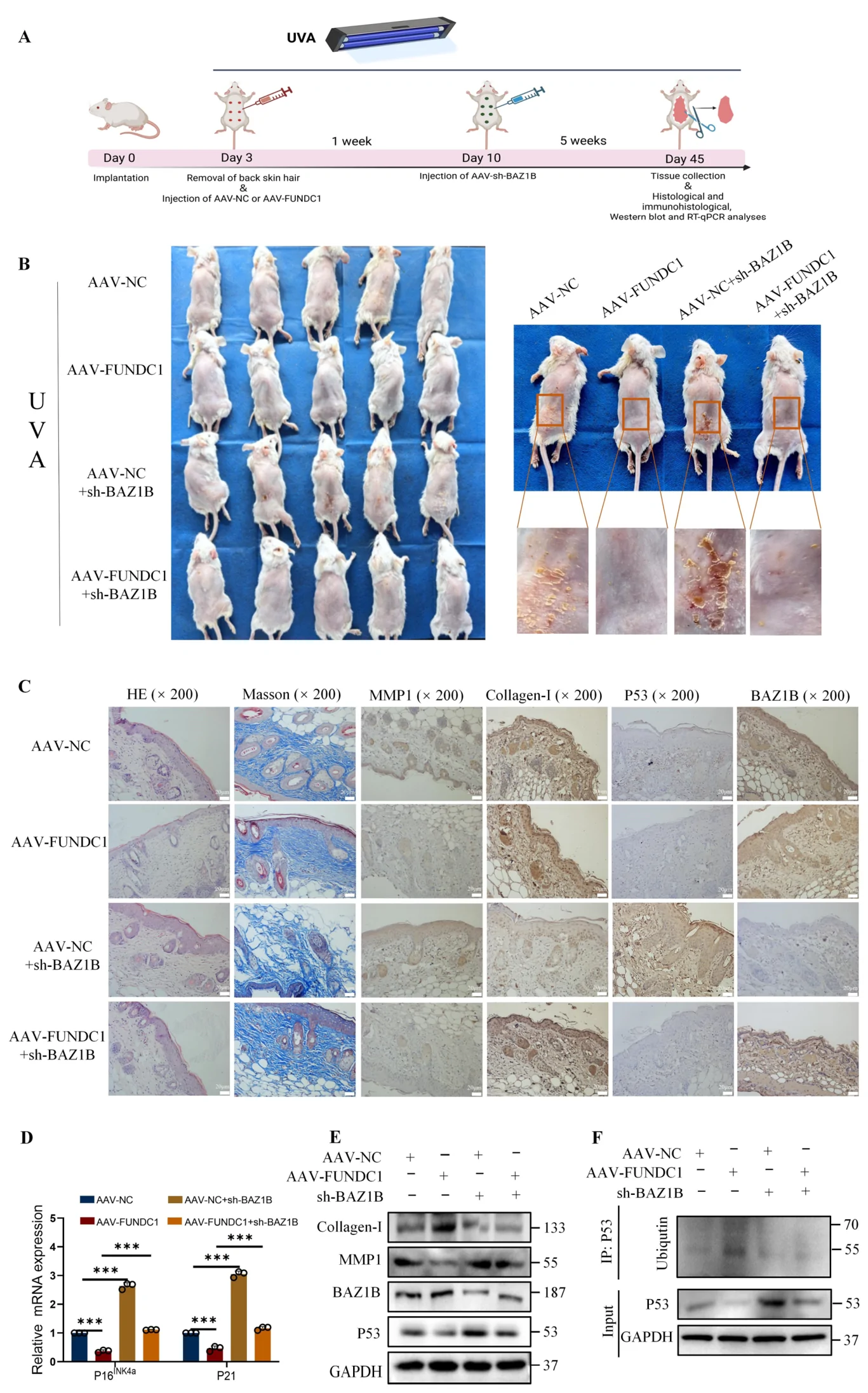
BAZ1B knockdown attenuates FUNDC1-mediated protection against UVA-induced skin photoaging in vivo. (**A**) Schematic illustration of the in vivo experimental design. Mice were treated with AAV-NC or AAV-FUNDC1, followed by AAV-sh-BAZ1B administration under UVA-induced photoaging conditions. (**B**) Representative gross images of dorsal skin and enlarged views of the treated skin area from AAV-NC, AAV-FUNDC1, AAV-NC + sh-BAZ1B, and AAV-FUNDC1 + sh-BAZ1B groups. (**C**) Hematoxylin and eosin staining, Masson’s trichrome staining, and immunohistochemical staining of MMP1, Collagen-I, P53, and BAZ1B in mouse skin sections. (**D**) RT-qPCR analysis of P16^INK4a^ and P21 mRNA expression in mouse skin tissues. (**E**) Western blot analysis of Collagen-I, MMP1, BAZ1B, and P53 expression. GAPDH was used as the loading control. (**F**) Immunoprecipitation analysis of P53 ubiquitination in mouse skin tissues. P53 was immunoprecipitated, and ubiquitinated P53 was detected by immunoblotting with an anti-ubiquitin antibody. Gross morphological assessment, histological staining, and immunohistochemical staining were performed using five mice per group (n = 5), and representative images are shown in panels (**B**,**C**). RT-qPCR, Western blot, and immunoprecipitation analyses were performed using skin tissues from three independent mice per group (n = 3). Data are presented as the mean ± SD. Data in panel (**D**) were analyzed using two-way ANOVA followed by Tukey’s multiple-comparisons test. Scale bars are indicated in the images. *** *p* < 0.001.

To further figure out whether BAZ1B contributes to FUNDC1-associated regulation of P53 ubiquitination in vivo, a mouse UVA-induced photoaging model was established as shown in Figure 10A. After six weeks of treatment, distinct morphological differences were observed among the experimental groups. Compared with the AAV-NC group, mice treated with AAV-FUNDC1 exhibited smoother dorsal skin, reduced epidermal thickening, and more densely organized dermal collagen fibers. Conversely, AAV-NC + sh-BAZ1B treatment aggravated photoaging-like alterations, including rougher skin texture, epidermal hyperplasia, and disrupted collagen architecture. Notably, the protective effects caused by FUNDC1 overexpression were markedly attenuated by concomitant BAZ1B knockdown, as reflected by increased epidermal thickness and collagen disorganization in the AAV-FUNDC1 + sh-BAZ1B group compared with the AAV-FUNDC1 group (Figure 10B,C).

Molecular analyses further supported these histological observations. FUNDC1 overexpression reduced the mRNA levels of the senescence-associated markers P16^INK4a^ and P21, whereas BAZ1B knockdown increased their expression. The inhibitory effects of FUNDC1 on P16^INK4a^ and P21 were partially reversed when BAZ1B was simultaneously silenced (Figure 10D). Immunohistochemical and Western blot analyses showed that FUNDC1 overexpression enhanced Collagen-I expression and attenuated MMP1 and P53 expression, together with elevated BAZ1B levels. These changes were weakened in the AAV-FUNDC1 + sh-BAZ1B group, indicating that BAZ1B knockdown compromised the protective effects resulting from FUNDC1 overexpression (Figure 10C,E).

Finally, immunoprecipitation assays showed that FUNDC1 overexpression increased P53 ubiquitination in photoaged skin, whereas BAZ1B knockdown reduced this enhancement in the AAV-FUNDC1 + sh-BAZ1B group (Figure 10F). Together, these in vivo findings suggest that BAZ1B is required, at least in part, for FUNDC1-related protection against UVA-induced skin photoaging and for the regulation of P53 ubiquitination in vivo.

## 4. Discussion

The present study identifies FUNDC1 as a protective regulator in UVA-induced skin photoaging. In HDFs and mouse skin, UVA exposure reduced FUNDC1 expression and was accompanied by mitochondrial dysfunction, altered autophagy/mitophagy-related signaling, and photoaging-associated changes. FUNDC1 loss exacerbated these phenotypes, whereas FUNDC1 overexpression alleviated them, supporting a role for FUNDC1-associated mitochondrial quality control. Mechanistically, miR-137-3p negatively regulated FUNDC1 through the FUNDC1 3′UTR. In addition to mitochondrial effects, FUNDC1 was linked to post-transcriptional regulation of P53 stability through a proteasome-dependent process. BAZ1B was identified as a candidate regulator involved in FUNDC1-associated regulation of P53 ubiquitination and stability, and P53 K292 was implicated as a major site associated with BAZ1B-related regulation of P53 turnover. These findings provide an integrated framework connecting miR-137-3p/FUNDC1, mitochondrial homeostasis, and BAZ1B/P53 signaling in UVA-induced skin photoaging.

UVA-induced skin photoaging is closely associated with mitochondrial dysfunction and oxidative stress [15,22,23]. Because damaged mitochondria can amplify ROS accumulation and energy deficiency, efficient mitochondrial quality control is essential for maintaining fibroblast homeostasis under UVA stress [24]. As a mitochondrial outer membrane mitophagy receptor, FUNDC1 has been implicated in stress-related mitochondrial quality control [25,26]. However, the role of mitophagy in skin photoaging appears to be context-dependent. Some studies suggest that enhanced mitophagy can restore mitochondrial function and alleviate UVA-induced skin photoaging [27], whereas others indicate that excessive or dysregulated mitophagy may contribute to cellular injury or apoptosis under certain conditions. These observations reflect that the biological consequence of mitophagy may depend on the extent, duration and cellular context of mitochondrial stress. In the present study, FUNDC1 deficiency aggravated mitochondrial dysfunction and senescence-associated phenotypes, whereas FUNDC1 replenishment improved mitochondrial homeostasis and attenuated photoaging-associated changes. Pharmacological modulation with Rapa and Mdivi-1 further supported the involvement of mitophagy-related processes, although these compounds may affect additional cellular pathways. Thus, these findings should be interpreted as evidence that FUNDC1-associated mitophagy-related mitochondrial quality control contributes to protection against UVA-induced photoaging, rather than as proof of an exclusively FUNDC1-dependent mitophagy mechanism.

Extensive research has demonstrated that microRNAs, as key non-coding RNAs, participate in diverse biological processes, including cellular homeostasis, proliferation, differentiation, aging, and energy metabolism [28]. Their roles in skin development, homeostasis maintenance, and dermatological disorders have also attracted growing attention [29,30,31]. Microarray analysis of UVA-irradiated photoaged HDFs identified 146 upregulated and 4 downregulated miRNAs [32]. For instance, miR-152 has been shown to promote cellular senescence by directly targeting integrin alpha 5 (ITGα5), thereby suppressing HDF proliferation, while ITGα5 itself mediates fibronectin receptor-dependent signal transduction essential for cell adhesion and migration [33]. In the current study, bioinformatic prediction followed by dual-luciferase reporter assays supported FUNDC1 as a direct target of miR-137-3p. miR-137-3p was upregulated in UVA-induced photoaging models, and the miR-137-3p mimic reduced FUNDC1 expression, impaired mitophagy-related activity, increased ROS accumulation, decreased ATP production, and promoted photoaging-associated phenotypes. Conversely, miR-137-3p inhibition increased FUNDC1 expression and attenuated these changes under UVA-induced photoaging conditions. These findings suggest that miR-137-3p-mediated FUNDC1 downregulation may contribute to mitochondrial dysfunction and photoaging-associated cellular changes in HDFs. In addition, co-transfection of si-FUNDC1 with the miR-137-3p inhibitor partially restored FUNDC1 expression and partially counteracted FUNDC1 knockdown-associated changes. Notably, si-FUNDC1 targeted the coding region of FUNDC1, whereas miR-137-3p regulates FUNDC1 through its 3′UTR. Because siRNA-mediated knockdown did not completely eliminate endogenous FUNDC1 expression, inhibition of miR-137-3p may relieve repression of the residual FUNDC1 transcripts, thereby accounting for the partial restoration of FUNDC1 expression observed in the co-transfection experiments. Nevertheless, because miR-137-3p may also regulate additional targets, the contribution of other miR-137-3p-mediated pathways to the partial phenotypic rescue cannot be excluded. Further studies using reciprocal gain- and loss-of-function rescue approaches would help to strengthen this regulatory relationship. Recently, emerging therapeutic strategies have explored exosome-mediated miRNA delivery for skin disease treatment [34,35]. The unique lipid bilayer structure of exosomes, mirroring that of donor cells, enables efficient intercellular communication across physiological barriers [36,37]. Although the present study did not investigate exosome-based delivery of miR-137-3p, this approach represents a promising avenue for future exploration in photoaging intervention.

After establishing the functional involvement of FUNDC1 in photoaging-associated mitochondrial dysfunction, we further examined P53 signaling because P53 is closely associated with cellular senescence, stress responses, and mitochondrial homeostasis [38,39,40]. FUNDC1 manipulation altered P53 protein levels without markedly affecting P53 mRNA expression, and CHX chase analysis together with MG132 and Baf-A1 treatment suggested that FUNDC1 overexpression was associated with proteasome-dependent P53 turnover [41]. These findings indicate that FUNDC1 may influence P53 stability through a post-translational mechanism. The biological consequence of this regulation should be interpreted in a context-dependent manner. In UVA-induced photoaged fibroblasts, persistent P53 activation may contribute to senescence-associated growth arrest and matrix remodeling; therefore, limiting excessive P53 accumulation may be involved in the protective effects associated with FUNDC1. However, P53 also plays essential roles in DNA-damage surveillance and tumor-suppressive responses, particularly under UV stress. Thus, FUNDC1-associated reduction in P53 protein should not be interpreted as a general therapeutic goal, but rather as a context-specific regulatory event in the UVA-induced photoaging model examined in this study.

The ubiquitin-proteasome system involves the coordinated actions of E1, E2 and E3 enzymes, among which E3 ligases largely determine substrate specificity [42]. To explore potential ubiquitination regulators involved in P53 stability, candidate P53-associated E3 ligases were predicted using UbiBrowser. Among the predicted candidates, BAZ1B showed a high predicted interaction score with P53 and was selected for further validation. Co-immunoprecipitation and fluorescence colocalization analyses revealed an association between BAZ1B and P53. Functional studies showed that BAZ1B knockdown increased P53 protein levels and aggravated photoaging-associated phenotypes, whereas BAZ1B overexpression reduced P53 protein expression and promoted P53 protein turnover. Moreover, FUNDC1 repletion was associated with increased BAZ1B protein expression and enhanced P53 ubiquitination, while BAZ1B knockdown attenuated FUNDC1-associated P53 ubiquitination. These findings support the notion that BAZ1B participates in FUNDC1 regulation of P53 ubiquitination and stability. However, whether BAZ1B directly catalyzes P53 ubiquitination requires further biochemical validation, such as in vitro ubiquitination assays and catalytically inactive BAZ1B mutants. Therefore, the present data support BAZ1B as a candidate regulator involved in P53 ubiquitination and stability, but do not conclusively establish BAZ1B as the direct E3 ligase responsible for P53 ubiquitination.

Mass spectrometric analysis identified K120, K292, and K305 as candidate ubiquitination sites on P53. Among these sites, K292 appeared to be particularly relevant to BAZ1B-associated P53 regulation. Mutation of K292 to arginine reduced BAZ1B-associated P53 ubiquitination more evidently than K120R or K305R, and CHX chase analysis showed that P53K292R was less sensitive to BAZ1B-associated turnover than wild-type P53. These findings suggest that K292 is a major site associated with BAZ1B-related regulation of P53 ubiquitination and stability, rather than the sole or exclusive ubiquitination site involved in this process.

This finding can be interpreted in the broader context of P53 ubiquitination. P53 degradation is regulated by multiple E3 ligases acting on distinct lysine residues, forming a complex and context-dependent regulatory network. In addition to the canonical regulator MDM2, recent studies have identified additional ligases, such as CRL4B, which ubiquitinates P53 at K164 to promote P53 degradation in acute kidney injury, and FBXO33, which targets K291 and K292 to regulate P53 stability in response to metabolic signals [43,44]. In this context, K292 may represent an important lysine residue associated with BAZ1B-related regulation of P53 stability under UVA-induced stress. These observations raise the possibility that different E3 ligases may preferentially regulate specific lysine residues under distinct pathological conditions, thereby fine-tuning P53 activity in a stimulus- and tissue-dependent manner.

P53 ubiquitination is unlikely to function as an isolated event. In the context of skin photoaging, UVA-induced mitochondrial stress, ROS accumulation, metabolic alterations, and DNA damage responses may converge on P53 regulatory networks. For example, FBXO33-mediated ubiquitination of P53 at K291/K292 has been linked to metabolic regulation through YY1 lactylation [44], whereas CHK2-USP7 signaling can stabilize P53 during oxidative stress or DNA damage [45]. Therefore, the FUNDC1-associated BAZ1B/P53 axis proposed here should be viewed as a context-specific regulatory event that may interact with canonical DNA damage responses, oxidative stress signaling, and other senescence-associated pathways. Several limitations should be acknowledged. First, an important unresolved issue is how FUNDC1 is linked to BAZ1B regulation. Because FUNDC1 is a mitochondrial outer membrane protein, the present data do not support a simple direct regulatory mechanism between FUNDC1 and BAZ1B. Instead, FUNDC1-associated changes in BAZ1B abundance may involve indirect mitochondrial-to-nuclear signaling events, such as alterations in mitochondrial quality control, ROS-related signaling, metabolic status, transcriptional regulation, or BAZ1B protein stability. Further studies are required to define the precise molecular pathway connecting FUNDC1 to BAZ1B. Second, although BAZ1B was predicted as a candidate P53 ubiquitination regulator and contributed to enhanced P53 ubiquitination in our experimental system, additional biochemical evidence is needed to confirm whether BAZ1B directly catalyzes P53 ubiquitination. Third, although K292 was identified as a major site for BAZ1B-related P53 ubiquitination, P53 stability is regulated by multiple ubiquitination sites and E3 ligases; therefore, additional sites or regulators may also contribute to P53 turnover during UVA-induced photoaging. Fourth, pharmacological modulators such as Rapa and Mdivi-1 are useful tools but may have effects beyond mitophagy regulation. Genetic approaches targeting mitophagy-related machinery would further strengthen the conclusion that mitophagy-related activity contributes to FUNDC1-mediated protection. Finally, although the present study proposes a functional miR-137-3p/FUNDC1 relationship, additional miR-137-3p targets may also participate in photoaging-associated mitochondrial dysfunction.

In summary, our study supports a regulatory model in which UVA-induced photoaging is accompanied by miR-137-3p upregulation, FUNDC1 downregulation, impaired mitophagy-related mitochondrial quality control, and altered BAZ1B/P53 signaling. FUNDC1 appears to alleviate photoaging and associated mitochondrial dysfunction, at least in part, through mitophagy-related mechanisms and BAZ1B-associated regulation of P53 ubiquitination and stability. These findings provide a framework linking miR-137-3p, FUNDC1, mitochondrial homeostasis, and BAZ1B/P53 signaling in skin photoaging and highlight the need for further studies to define the precise molecular connections between mitochondrial quality control and nuclear P53 regulatory networks.

## 5. Conclusions

In conclusion, the present study demonstrates that FUNDC1 is downregulated in UVA-induced photoaged HDFs and mouse skin and that FUNDC1 alleviates photoaging-associated mitochondrial dysfunction and senescence-like changes. Mechanistically, miR-137-3p negatively regulates FUNDC1 expression through the FUNDC1 3′UTR. FUNDC1 is associated with mitophagy-related mitochondrial quality control and BAZ1B-related regulation of P53 ubiquitination and stability. In addition, P53 K292 was identified as a major ubiquitination site associated with BAZ1B-related regulation of P53 turnover. Together, these findings provide a regulatory framework linking miR-137-3p/FUNDC1, mitochondrial homeostasis, and BAZ1B/P53 signaling in UVA-induced skin photoaging, while the precise molecular connection between FUNDC1 and BAZ1B remains to be further defined.

## Figures and Tables

**Table 1:** Primers for RT-qPCR.

Gene	Accession No.	Forward	Reverse
β-actin	NM_001101.5	GCATCCACGAGACCACCTACAAC	AGCCACCGATCCAGACAGAGTAC
P16^INK4a^	NM_000077.5	AGCCACCGATCCAGACAGAGTAC	CGGGGATGTCTGAGGGACCTTC
P21	NM_000389.5	GATGTCCGTCAGAACCCATGCG	CCTGCCTCCTCCCAACTCATCC
BAZ1B	NM_032408.3	TCTGATGGTGCCTGTGATTCTCC	TGTCTCCTTCTTCTGATGGTCCTG
P53	NM_000546.6	CCTCCTCAGCATCTTATCCGAGTG	CCAACCTCAGGCGGCTCATAG

## Data Availability

The data that support the findings of this study are available from the corresponding author upon reasonable request due to privacy and ethical restrictions.

## Supplementary Materials

The following supporting information can be downloaded at: https://www.mdpi.com/article/10.3390/antiox15091063/s1, Supplementary Figure S1. UVA exposure induces morphological changes and reduces proliferative activity in HDFs; Supplementary Figure S2. Validation of FUNDC1 knockdown and overexpression efficiency in HDFs; Supplementary Figure S3. MiR-137-3p regulates senescence-associated marker expression and ATP production in HDFs; Supplementary Figure S4. MiR-137-3p inhibition partially counteracts the changes in HDFs caused by FUNDC1 knockdown; Supplementary Figure S5. BAZ1B knockdown validation and altered BAZ1B/P53 signaling in UVA-induced photoaged skin; Supplementary Figure S6. Isolation and characterization of primary human dermal fibroblasts.
