# Supplementary material for: FUNDC1 Attenuates UVA-Induced Skin Photoaging by Regulating Mitophagy and P53 Stability
Source: Antioxidants (Basel). 2026 Aug 25;15(9):1063. doi: 10.3390/antiox15091063 (PMC13603441; doi:10.3390/antiox15091063)
Supplement: Supplementary file 1 [file antioxidants-15-01063-s001.zip › antioxidants-4474786-supplementary.pdf]

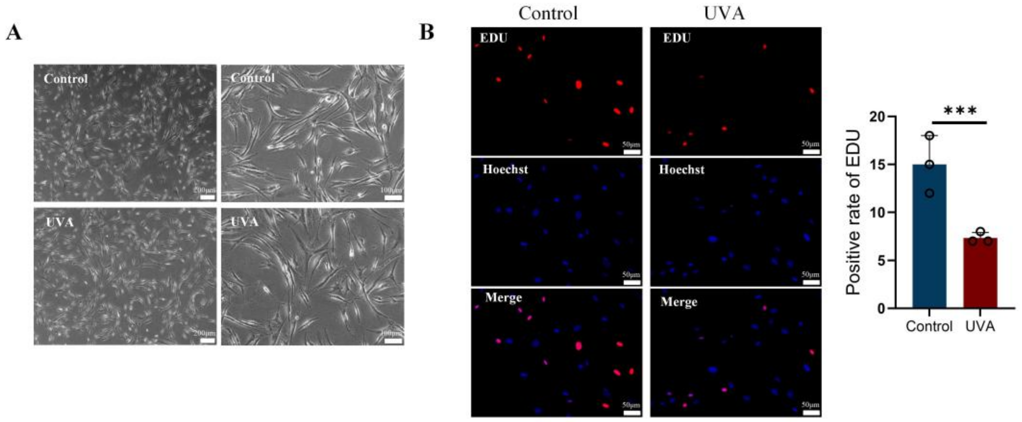

Supplementary Figure S1. UVA exposure induces morphological changes and reduces proliferative activity in HDFs.

(A) Representative phase-contrast images of control and UVA-exposed HDFs showing morphological alterations after UVA irradiation. (B) Representative EdU staining images and quantification of EdU-positive cells in control and UVA-exposed HDFs. Hoechst staining was used to label nuclei. Data are presented as mean ± SD from three independent biological replicates ($n = 3$). Statistical significance was determined by unpaired two-tailed Student's t-test. Scale bars are indicated in the images. ***$p < 0.001$.

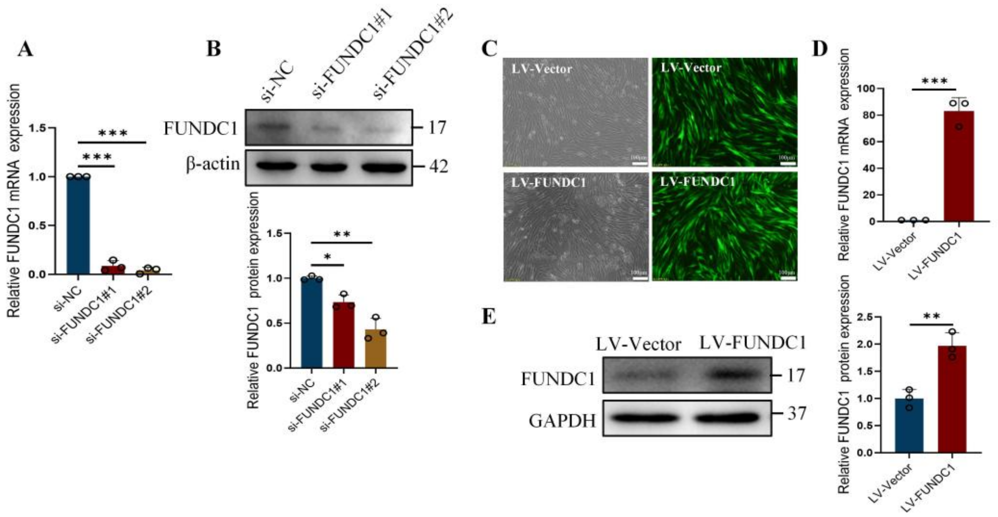

Supplementary Figure S2. Validation of FUNDC1 knockdown and overexpression efficiency in HDFs. (A) RT-qPCR analysis of FUNDC1 mRNA expression in HDFs transfected with si-NC, si-FUNDC1#1, or si-FUNDC1#2. (B) Western blot analysis and quantification of FUNDC1 protein expression after FUNDC1 knockdown. β-actin was used as the loading control. (C) Representative bright-field and fluorescence images showing lentiviral transduction efficiency in LV-Vector and LV-FUNDC1 groups. (D) RT-qPCR analysis of FUNDC1 mRNA expression after FUNDC1 overexpression. (E) Western blot analysis and quantification of FUNDC1 protein expression after FUNDC1 overexpression. GAPDH was used as the loading control. Data are presented as mean ± SD from three independent biological replicates ($n = 3$). Data in S2A and S2B were analyzed using one-way ANOVA followed by Tukey's multiple-comparisons test. Data in S2D and S2E were analyzed using unpaired two-tailed Student's t-tests. Scale bars are indicated in the images. *$p < 0.05$, **$p < 0.01$, ***$p < 0.001$.

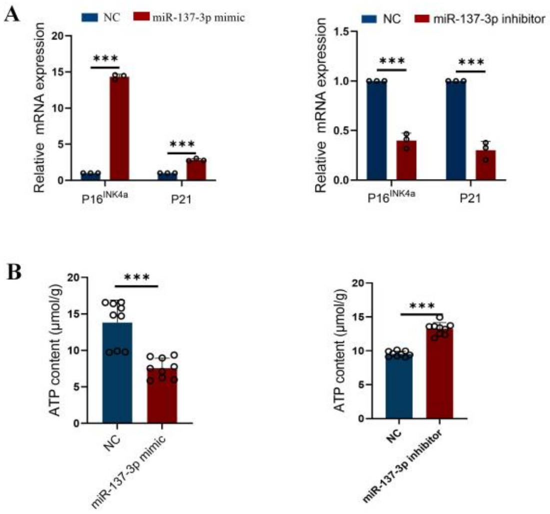

Supplementary Figure S3. MiR-137-3p regulates senescence-associated marker expression and ATP production in HDFs.

A) RT-qPCR analysis of $P16^{INK4a}$ and P21 mRNA expression after transfection with miR-137-3p mimic or miR-137-3p inhibitor. (B) ATP content after miR-137-3p mimic or miR-137-3p inhibitor transfection. For gain-of-function analysis, HDFs were transfected with miR-137-3p mimic. For loss-of-function analysis, UVA-exposed HDFs were transfected with miR-137-3p inhibitor. Data are presented as mean ± SD from three independent biological replicates (n = 3). Statistical significance was determined by unpaired two-tailed Student's t-test. ***$p < 0.001$.

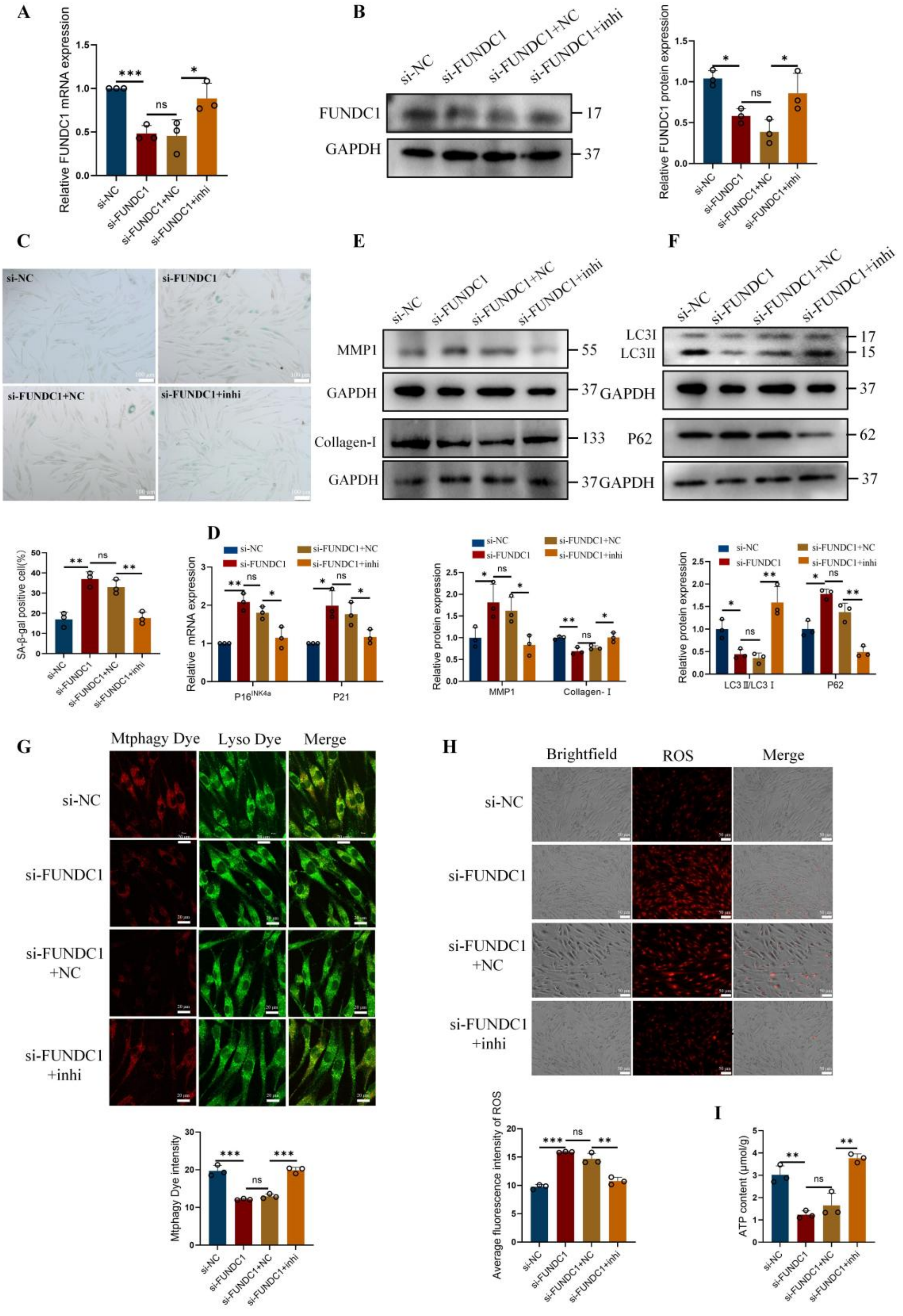
A
B
C
D
E
F
G
H
I
Relative FUNDC1 mRNA expression
Relative FUNDC1 protein expression
FUNDC1
GAPDH
si-NC
si-FUNDC1
si-FUNDC1+NC
si-FUNDC1+inhi
MMP1
Collagen-I
LC3I
LC3II
P62
SA-β-gal positive cell(%)
Relative mRNA expression
Relative protein expression
Mtphagy Dye
Lyso Dye
Merge
Brightfield
ROS
Mtphagy Dye intensity
Average fluorescence intensity of ROS
ATP content (μmol/g)

Supplementary Figure S4. MiR-137-3p inhibition partially counteracts the changes in HDFs caused by FUNDC1 knockdown.

(A) RT-qPCR analysis of FUNDC1 mRNA expression in HDFs transfected with si-NC, si-FUNDC1, si-FUNDC1+NC inhibitor, or si-FUNDC1+miR-137-3p inhibitor. (B) Western blot analysis and quantification of FUNDC1 protein expression. (C) Representative SA-β-gal staining images and quantification of SA-β-gal-positive cells. (D) RT-qPCR analysis of $P16^{INK4a}$ and P21 mRNA expression. (E) Western blot analysis and quantification of MMP1 and Collagen-I expression. GAPDH was used as the loading control. (F) Western blot analysis and quantification of LC3-II/LC3-I and P62 expression. GAPDH was used as the loading control. (G) Representative Mtphagy Dye and Lyso Dye staining images and quantification of Mtphagy Dye fluorescence intensity. (H) Representative DHE staining images and quantification of intracellular ROS fluorescence intensity. (I) ATP content in the indicated treatment groups. Data are presented as mean ± SD from three independent biological replicates (n = 3). Statistical significance was determined by one-way ANOVA followed by Tukey's multiple-comparisons test. Scale bars are indicated in the images. ns, not significant; $*p < 0.05$, $**p < 0.01$, $***p < 0.001$.

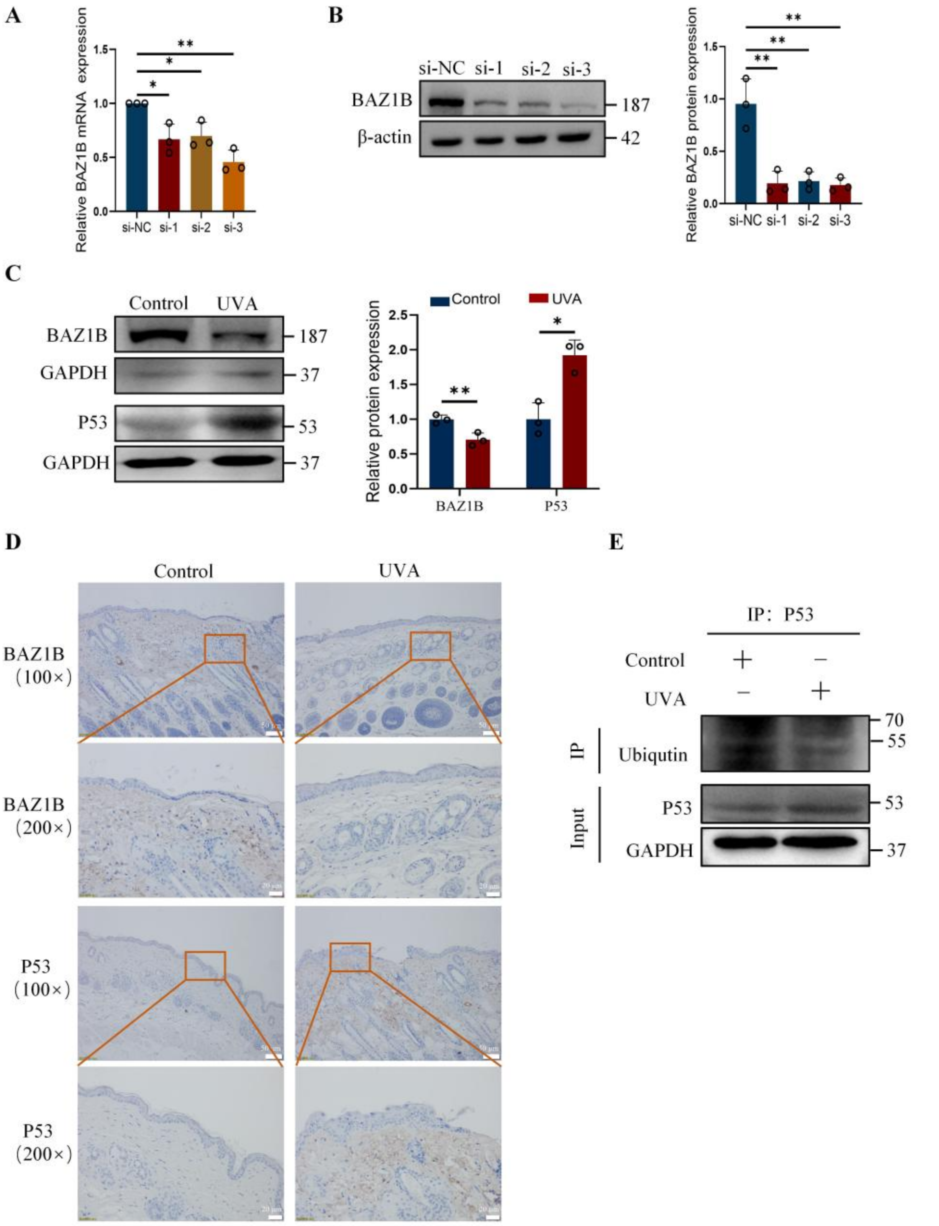

Supplementary Figure S5. BAZ1B knockdown validation and altered BAZ1B/P53 signaling in UVA-induced photoaged skin.

(A) RT-qPCR analysis of BAZ1B mRNA expression in HDFs transfected with si-NC or three independent BAZ1B siRNAs. (B) Western blot analysis and quantification of BAZ1B protein expression after BAZ1B knockdown. β-actin was used as the loading control. (C) Western blot analysis and quantification of BAZ1B and P53 protein expression in control and UVA-irradiated mouse skin tissues. GAPDH was used as the loading control. (D) Representative immunohistochemical staining of BAZ1B and P53 in control and UVA-irradiated mouse skin sections. (E) Immunoprecipitation analysis of P53 ubiquitination in control and UVA-irradiated mouse skin tissues. P53 was immunoprecipitated, and ubiquitinated P53 was detected by immunoblotting with an anti-ubiquitin antibody. Quantitative cell-based and mouse tissue analyses were performed using three independent biological replicates (n = 3), whereas immunohistochemical staining was performed using five mice per group (n = 5), with representative images shown. Data are presented as mean ± SD. Statistical significance was determined by unpaired

two-tailed Student's t-test or one-way ANOVA followed by Tukey's multiple-comparisons test, as appropriate. Scale bars are indicated in the images. *$p < 0.05$, **$p < 0.01$.

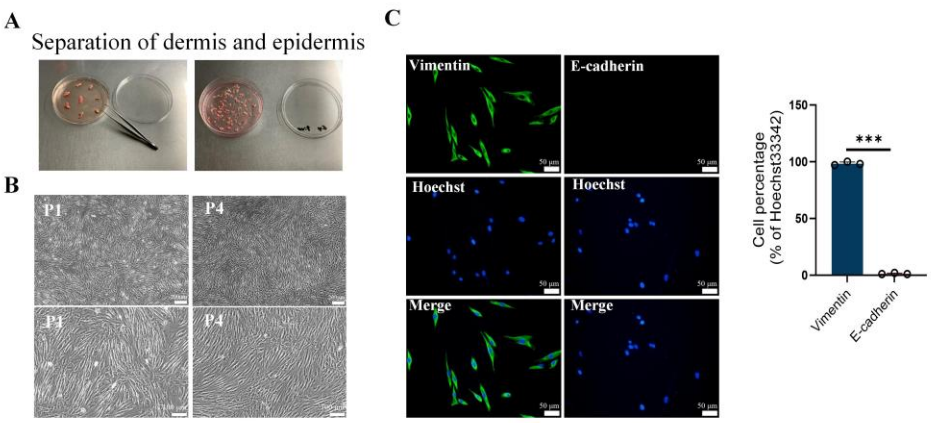

Supplementary Figure S6. Isolation and characterization of primary human dermal fibroblasts.

(A) Representative images showing separation of dermis and epidermis during primary human dermal fibroblast isolation. (B) Representative phase-contrast images of primary dermal fibroblasts at passage 1 (P1) and passage 4 (P4). (C) Representative immunofluorescence staining of Vimentin and E-cadherin in cultured primary human dermal fibroblasts, with Hoechst staining used to label nuclei. Quantitative data in panel C are presented as mean ± SD from three independent donor-derived HDF preparations ($n = 3$). Statistical significance in panel S6C was determined using an unpaired two-tailed Student's t-test. Scale bars are indicated in the images. ***$p < 0.001$.
